# Superior diagnostic efficacy and cost advantage of FibroTouch over CHI3L1 for liver fibrosis in Chinese patients with chronic liver disease

**DOI:** 10.1371/journal.pone.0357883

**Published:** 2026-10-05

**Authors:** Zhongbao Zuo, Chunli Yang, Lanlan Hu, Xiafen Ye, Yazhen Zhang, Lili Huang, Miaochan Wang, Huaizhong Cui, Xiaoqiang Chen, Kenv Pan, Jianfeng Bao, He He

**Affiliations:** 1 Department of Clinical Laboratory, Hangzhou Xixi Hospital, Hangzhou Sixth People’s Hospital, Hangzhou Xixi Hospital Affiliated to Zhejiang Chinese Medical University, Hangzhou, Zhejiang, China; 2 Department of Clinical Laboratory, The 903rd Hospital of PLA, Hangzhou, Zhejiang, China; 3 Department of Hepatology, Hangzhou Xixi Hospital, Hangzhou Sixth People’s Hospital, Hangzhou Xixi Hospital Affiliated to Zhejiang Chinese Medical University, Hangzhou, Zhejiang, China; 4 Department of Stomatology, Hangzhou Xixi Hospital, Hangzhou Sixth People’s Hospital, Hangzhou Xixi Hospital Affiliated to Zhejiang Chinese Medical University, Hangzhou, Zhejiang, China; Johns Hopkins University School of Medicine, UNITED STATES OF AMERICA

## Abstract

**Background:**

Non-invasive assessment of liver fibrosis is essential for managing chronic liver disease (CLD). Serum chitinase-3-like protein 1 (CHI3L1) has been recommended in recent guidelines, while FibroTouch is a domestically produced device for liver stiffness measurement (LSM). However, no study has directly compared their diagnostic performance in CLD patients.

**Methods:**

This retrospective study included 235 patients with CLD who underwent liver biopsy, FibroTouch examination, and laboratory testing (including CHI3L1 and routine blood parameters) at Hangzhou Xixi Hospital between October 2019 and December 2024. Liver fibrosis was staged according to the Scheuer scoring system (S0–S4). The diagnostic performance of LSM, CHI3L1, and six serological models (aspartate aminotransferase-to-platelet ratio index [APRI], fibrosis-4 index [FIB-4], S-Index, Forns index, platelet ratio-to-RDW [PRP], and FIB-5) were evaluated using receiver operating characteristic (ROC) curve analysis, with liver biopsy as the reference standard.

**Results:**

Among the 235 patients, 125 (53.2%) had significant fibrosis (≥S2). LSM demonstrated excellent diagnostic accuracy, with area under the curve (AUROC) values of 0.86 for detecting significant fibrosis (≥S2), 0.91 for advanced fibrosis (≥S3), and 0.93 for cirrhosis (S4). CHI3L1 showed moderate diagnostic performance, with corresponding AUROC values of 0.72, 0.79, and 0.81. LSM exhibited the strongest correlation with histological staging (r = 0.70, p < 0.0001). Among serological models, APRI and FIB‑4 showed relatively better performance, while FIB‑5 performed poorly.

**Conclusions:**

In this biopsy-proven CLD cohort, LSM demonstrated superior diagnostic performance for liver fibrosis assessment compared to CHI3L1 and six serological models, with the strongest correlation with histological staging. Given its better diagnostic efficacy and lower cost, LSM offers a practical advantage as a routine monitoring tool in clinical practice, particularly in resource-constrained settings.

## 1. Introduction

The global burden of chronic liver disease (CLD) has remained substantial over the last decades [1,2]. While the prevalence and incidence of hepatitis B and C have declined, those of metabolic-associated steatohepatitis (MASLD) and other CLD have increased [1]. China had 385.6 million cases of CLD in 2021 [2], but 74.6% of them were MASLD. CLD typically advance through distinct phases, starting with hepatitis, progressing to liver fibrosis, and eventually leading to cirrhosis and hepatocellular carcinoma. Among these stages, liver fibrosis is a crucial indicator of disease progression and has a substantial impact on clinical outcomes for patients with CLD. Therefore, routine monitoring of liver fibrosis in chronic liver disease is particularly important.

Currently, liver biopsy remains the “gold standard” for diagnosing liver fibrosis. However, its invasive nature, potential for observer and sampling errors, and the associated trauma make it impractical for widespread use in real-world clinical settings. FibroTouch, a domestically produced device in China, has shown promising clinical efficacy. It assesses the degree of liver fibrosis by measuring liver stiffness (LSM). Our previous research [3] has indicated that FibroTouch is an effective tool with high sensitivity and specificity. Several serological models have been developed for diagnosing liver fibrosis, including the aspartate transaminase-to-platelet ratio index (APRI) [4], FIB-4 [5], S-Index [6], Forns [7], PRP [8], and Fib-5 [9]. While these models utilize routine blood tests to assess liver fibrosis, their diagnostic accuracy is often suboptimal and requires cautious interpretation, particularly at the individual patient level.

Serum chitinase-3-like protein 1 (CHI3L1, also known as YKL-40) belongs to the mammalian chitinase family [10]. Under physiological conditions, CHI3L1 is expressed at low basal levels; however, its expression is markedly upregulated in response to pro inflammatory stimuli (e.g., TNF α, IL 6), tissue hypoxia, and ongoing extracellular matrix (ECM) remodeling [10]. Mechanistically, CHI3L1 contributes to liver fibrogenesis by binding to the interleukin 13 receptor α2 (IL 13Rα2) on hepatic stellate cells (HSCs), thereby activating downstream MAPK/ERK and PI3K/AKT signaling cascades [10]. This activation promotes HSCs proliferation, migration, and survival, while simultaneously suppressing their apoptosis, ultimately driving the accumulation of ECM and progressive fibrous septa formation [10]. Consequently, elevated serum CHI3L1 levels not only reflect the degree of inflammatory activity but also directly participate in the pathological progression of fibrosis. Research [11–13] has shown that patients with liver fibrosis exhibit significantly elevated serum CHI3L1 levels compared to healthy individuals, and that these heightened levels are associated with more severe fibrosis. This evidence points to CHI3L1’s significant involvement in liver fibrosis. Moreover, serum CHI3L1 demonstrates a strong correlation with transient elastography and aligns well with liver histopathology [14]. Recognized for its noninvasive diagnostic potential in liver fibrosis, serum CHI3L1 has been endorsed at international hepatology conferences and incorporated into the Chinese Society of Hepatology’s “Guidelines for the prevention and treatment of chronic hepatitis B (version 2022)” [15].

Studies have shown that the diagnostic efficacy (AUROC) of CHI3L1 for liver fibrosis in chronic liver diseases ranges from 0.76 to 0.97 [12,13,16,17], while that of FibroTouch ranges from 0.71 to 0.92 [18–20]. Although CHI3L1 has demonstrated higher diagnostic efficacy in some studies, FibroTouch remains valuable in clinical practice due to its non-invasive nature and rapid testing capabilities. It can quickly assess the degree of liver fibrosis and is suitable for large-scale screening and clinical monitoring. In our previous work, we demonstrated that FibroTouch outperforms six serum-based models for liver fibrosis [3]. In the current study, we extend this comparison to include CHI3L1, with a particular focus on direct comparison between FibroTouch and CHI3L1—an area that remains unexplored in chronic liver diseases to date.

As China transitions into an aging society, the prevalence and incidence of chronic liver disease among the elderly population have been gradually increasing. Current healthcare reform policies in China require clinicians to achieve maximum medical benefits with minimal economic investment. In clinical practice, constraints on medical insurance reimbursement have created an increasing demand for clinicians to adopt accurate and cost-effective methods to monitor liver fibrosis risk in patients with CLD. Therefore, this study aims to evaluate the ability of CHI3L1, FibroTouch, and other non-invasive serum models to assess liver fibrosis in chronic liver diseases.

## 2. Materials and methods

### 2.1. Study populations

This retrospective study included patients with chronic liver disease who underwent liver biopsy in the Department of Hepatology at Hangzhou Xixi Hospital between October 1, 2019, and December 31, 2024. The inclusion criteria were: (1) age ≥ 18 years; (2) diagnosis of chronic liver disease; (3) underwent liver biopsy; (4) provided written informed consent. Patients were excluded if they had: (1) HIV co-infection; (2) any malignancy; (3) incomplete clinical data. A flowchart illustrating the patient enrollment process is shown in Fig 1. All participants underwent a FibroTouch examination within one week before or after the liver biopsy, and blood samples for routine laboratory tests were collected within one week prior to the procedure. This study was approved by the Institutional Ethics Review Committee of Hangzhou Xixi Hospital (Approval No: 2025001) and was conducted in accordance with the ethical principles outlined in the 1975 Declaration of Helsinki. Due to the retrospective nature of the study, the requirement for written informed consent was waived. After the ethics committee approved this study, we obtained the data for this study between 01/04/2026 and 30/06/ 2026.

**Fig 1:**
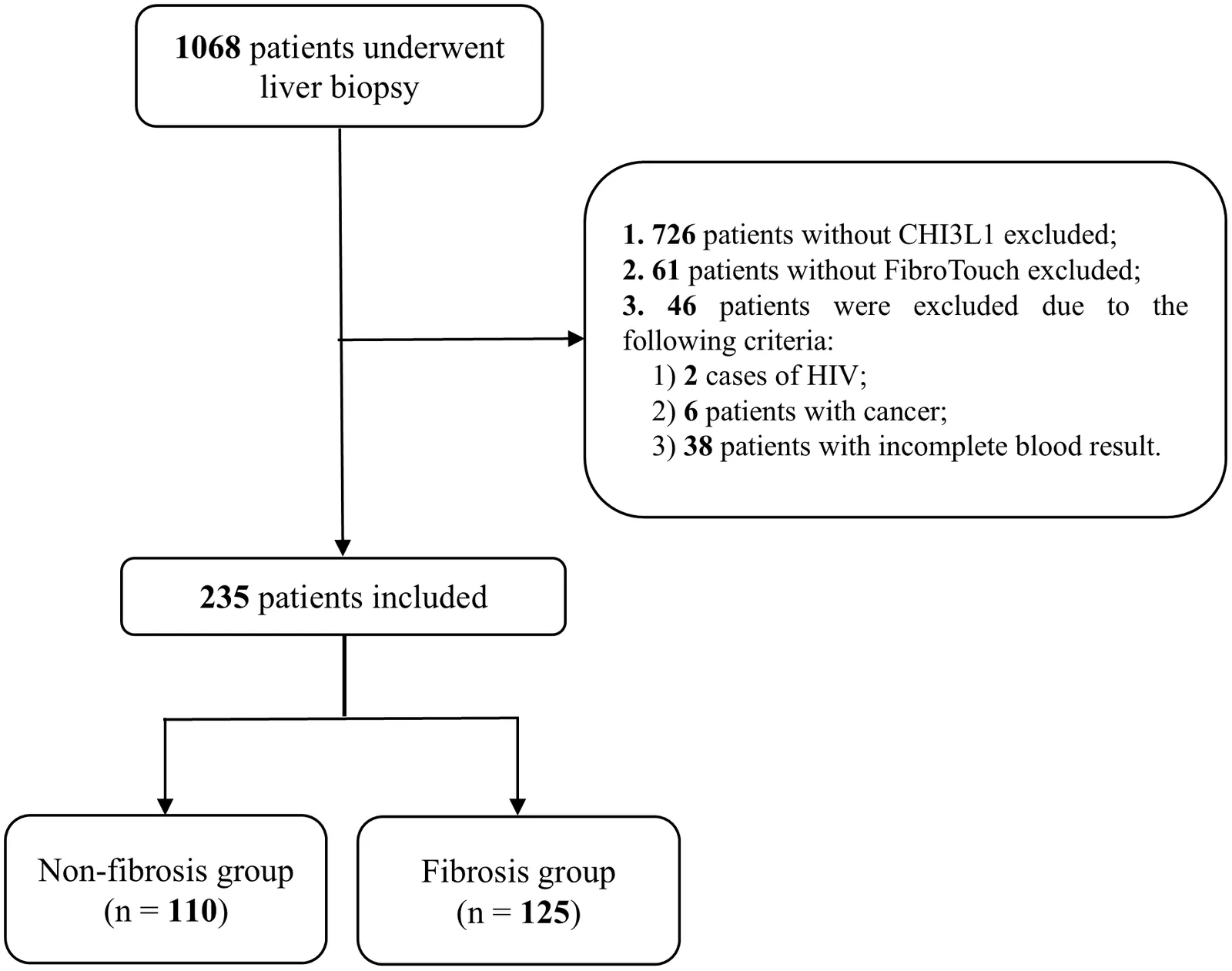
Flow chart of the study design and patient enrollment, 235 patients finally included in our research.

### 2.2. Blood test

Serum biochemical parameters, including alanine aminotransferase (ALT), aspartate aminotransferase (AST), gamma-glutamyl transferase (GGT), cholesterol (CHO), albumin (ALB), and alkaline phosphatase (ALP), were measured using the Beckman Coulter AU5831 automatic biochemical analyzer in the Department of Clinical Laboratory at Hangzhou Xixi Hospital. Hematological parameters, such as platelet count (PLT) and red blood cell distribution width (RDW), were determined using an SYSMEX automated hematology analyzer (XN9000).

### 2.3. FibroTouch

FibroTouch examinations were performed with the patient in the supine position, with the right arm fully abducted and placed behind the head to widen the intercostal spaces. Measurements were taken from the right anterior axillary line to the mid-axillary line, specifically targeting the 7th, 8th, and 9th intercostal spaces. The probe was positioned perpendicularly to the skin surface over an intercostal space. For each patient, the FibroTouch liver stiffness measurement (LSM) was performed, and the final result was reported as the median value of ten consecutive successful acquisitions. An examination was considered unreliable and excluded if the interquartile range (IQR) of the ten measurements exceeded one-third of the median value, or if the success rate was below 60%. All procedures were conducted by a single, trained operator to ensure consistency.

### 2.4. CHI3L1

Serum levels of CHI3L1 were measured using an enzyme-linked immunosorbent assay (ELISA) kit according to the manufacturer’s instructions. The kits were provided by Hangzhou Puwang Biotechnology Ltd. (Hangzhou, China).

### 2.5. Histopathological staging of liver fibrosis

Percutaneous liver biopsies were performed under ultrasound guidance using an automatic biopsy gun. All procedures were approved by the Ethics Committee of Hangzhou Xixi Hospital, and written informed consent was obtained from each patient prior to the biopsy. Liver tissue specimens, measuring 1.5 to 2.0 cm in length, were obtained and immediately fixed in 4% formaldehyde, then embedded in paraffin. Sections of 5 μm thickness were prepared and stained with hematoxylin and eosin (H&E) as well as Masson’s trichrome for histological evaluation. Only specimens containing at least six portal tracts were considered adequate for assessment.

Hepatic fibrosis was staged independently by two experienced pathologists in a double-blind manner using the Scheuer scoring system, as previously described [3,21]. The stages were defined as follows: S0, no fibrosis; S1, enlarged fibrotic portal tracts; S2, periportal fibrosis with septa formation but preserved lobular architecture; S3, fibrosis with lobular distortion without overt cirrhosis; and S4, probable or definite cirrhosis.

### 2.6. The calculation formula of serological model


$$\begin{array}{c} \text{APRI}=(\text{AST}/\text{ULN})\times \text{100}/\text{PLT}; \end{array}$$

$$\begin{array}{c} \text{FIB}-\text{4}=(\text{age}\times \text{AST})/\left(\text{PLT}\times \text{ALT}\right); \end{array}$$

$$\begin{array}{c} \text{S}-\text{Index}=(\text{1000}\times \text{GGT})/\left(\text{PLT}\times {\text{ALB}}^{\text{2}}\right); \end{array}$$

$$\begin{array}{c} \text{Forns}=\text{7}.\text{811}-\text{3}.\text{131}\times \text{ln}(\text{PLT})+\text{0}.\text{781}\times \text{ln}(\text{GGT})+\text{3}.\text{467}\times \text{ln}(\text{age})-\text{0}.\text{014}\times \text{CHO}; \end{array}$$

$$\begin{array}{c} \text{PRP}=\text{RDW}/\text{PLT}; \end{array}$$

$$\begin{array}{c} \text{Fib}-\text{5}=\text{0}.\text{3}\times \text{ALB}+\text{0}.\text{05}\times \text{PLT}-\text{0}.\text{014}\times \text{ALP}+\text{6}\times (\text{AST}/\text{ALT})+\text{14}. \end{array}$$

### 2.7. Statistical analysis

All statistical analyses were performed using R software (version 4.0.2, R Development Core Team, 2020) and GraphPad Prism (version 10.5 for Windows; GraphPad Software, San Diego, CA, USA). Continuous variables were expressed as mean ± standard deviation (SD) or median with interquartile range (IQR), depending on the normality of the distribution. Categorical variables were presented as frequencies and percentages. Group comparisons for categorical variables (e.g., sex) were conducted using the chi‑square test or Fisher’s exact test, as appropriate. For comparisons among multiple fibrosis stages (S0–S4), continuous variables with non‑normal distribution were compared using the Kruskal‑Wallis test followed by Dunn’s post‑hoc test with Bonferroni correction for pairwise comparisons. Normally distributed variables were analyzed using one-way analysis of variance (ANOVA) followed by Tukey’s HSD test. In Fig 2, only pairwise comparisons between S1 and each higher stage (S2, S3, S4) are shown for visual clarity. All tests were two‑sided, and p‑value < 0.05 was considered statistically significant after adjustment for multiple testing where applicable. Diagnostic performance was evaluated using receiver operating characteristic (ROC) curve analysis, with the area under the curve (AUROC) quantifying accuracy (higher values denote better discriminative ability). Pairwise comparisons of ROC curves across LSM, CHI3L1, and the six serological models were performed using DeLong’s test for correlated data, as implemented in the pROC package for R. Optimal cut-off values were determined using the Youden index (maximizing sensitivity + specificity – 1), and the corresponding sensitivity, specificity, positive predictive value (PPV), and negative predictive value (NPV) were calculated for each diagnostic test at each fibrosis threshold. A two-tailed p value < 0.05 was considered statistically significant for differences between AUROCs.

**Fig 2:**
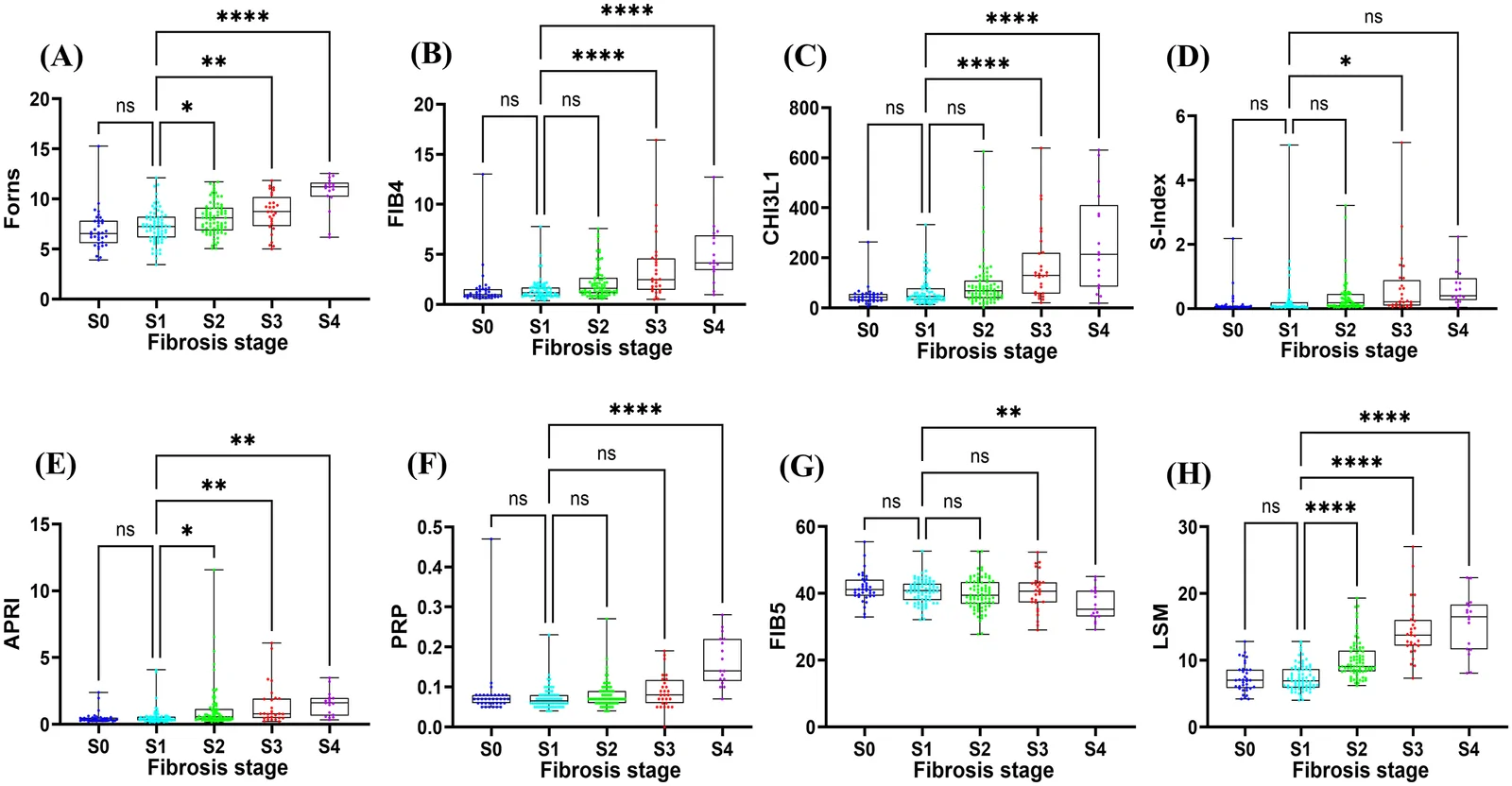
Non-invasive models, CHI3L1, and LSM levels among different fibrosis staging. (A) Forns; (B) FIB-4; (C) CHI3L1; (D) S-Index; (E) APRI; (F) PRP; (G) FIB5; (H) LSM. ns: not significant, *p < 0.05, **p < 0.01, ****p < 0.0001, by One-Way ANOVA test with Dunn’s multiple comparison test. CHI3L1, Chitinase 3-like protein 1; LSM, Liver stiffness measurement; APRI, Aspartate aminotransferase-to-platelet ratio index.

## 3. Results

### 3.1. General characteristics of the patients

A total of 235 patients with CLD who underwent liver biopsy, FibroTouch examination, and laboratory testing (including CHI3L1 and routine blood parameters) were included in the final analysis. Based on liver biopsy findings, 125 patients were classified as having moderate-to-severe fibrosis (fibrosis stage ≥ S2), including 44 males and 81 females with a median age of 53 years. The remaining 110 patients had no or mild fibrosis (stage < S2), comprising 50 males and 60 females with a median age of 47 years. No significant difference in sex distribution was observed between the two groups; however, age differed significantly across fibrosis stages.

Compared to patients with no or mild fibrosis, those with advanced fibrosis (stage ≥ S2) were significantly older and exhibited significantly higher levels of CHI3L1, RDW, ALT, AST, ALP, TBIL, DBIL, GGT, and LSM. Conversely, they had significantly lower PLT, ALB, and CHOL levels. In addition, all six serological fibrosis models showed significant differences across different fibrosis stages. Detailed comparisons are presented in Table 1.

**Table 1:** Characteristics of 235 patients with chronic liver disease in Zhejiang, China.

Variables	Total (n = 235)	Non-fibrosis (n = 110)	Fibrosis (n = 125)	P-value
Gender, n (%)				0.142
Male	94 (40.0)	50 (45.5)	44 (35.2)	
Female	141 (60.0)	60 (54.5)	81 (64.8)	
Age, Median (IQR)	50 (40, 56)	47 (38.25, 53)	53 (42, 60)	< 0.001
CHI3L1, Median (IQR)	57.38 (36.76, 114.25)	43.83 (31.37, 60.42)	83.41 (49.71, 143.23)	< 0.001
Diagnosis, n (%)				< 0.001
AIH	23 (9.8)	5 (4.5)	18 (14.4)	
CHB	135 (57.4)	78 (70.9)	57 (45.6)	
Cirrhosis	23 (9.8)	7 (6.4)	16 (12.8)	
OTHERS	54 (23)	20 (18.2)	34 (27.2)	
RDW, Median (IQR)	12.95 (12.5, 13.8)	12.75 (12.3, 13.2)	13.2 (12.7, 14.3)	< 0.001
PLT, Mean ± SD	183.1 ± 59.28	198 ± 52.86	169.98 ± 61.69	< 0.001
ALT, Median (IQR)	36 (21, 67.5)	33 (20.25, 49.25)	43 (21, 90)	0.007
AST, Median (IQR)	32 (25, 51.5)	27 (23, 38)	39 (27, 69)	< 0.001
ALP, Median (IQR)	89 (72, 119.5)	87 (70.5, 105.75)	95 (74, 134)	0.033
TP, Mean ± SD	70.38 ± 6.78	70.82 ± 6.07	69.99 ± 7.35	0.35
ALB, Mean ± SD	40.38 ± 4.64	41.93 ± 3.37	39.02 ± 5.16	< 0.001
TBIL, Median (IQR)	14.14 (11.2, 18.37)	13.04 (9.85, 16.55)	15.15 (12.21, 20.72)	< 0.001
DBIL, Median (IQR)	5.57 (4.17, 8.11)	4.76 (3.84, 5.92)	6.45 (4.82, 9.69)	< 0.001
CHOL, Median (IQR)	4.6 (4.04, 5.22)	4.84 (4.15, 5.34)	4.41 (3.88, 5.02)	0.014
GGT, Median (IQR)	34 (19.5, 83)	25 (16, 50.5)	51 (26, 103)	< 0.001
LSM, Median (IQR)	8.8 (6.9, 11.7)	6.95 (5.9, 8.7)	11.1 (8.9, 14.2)	< 0.001
APRI, Median (IQR)	0.48 (0.34, 0.92)	0.38 (0.29, 0.55)	0.71 (0.41, 1.56)	< 0.001
FIB-4, Median (IQR)	1.45 (0.94, 2.48)	1.1 (0.82, 1.66)	2.05 (1.26, 3.94)	< 0.001
S-Index, Median (IQR)	0.12 (0.06, 0.37)	0.07 (0.05, 0.15)	0.22 (0.09, 0.52)	< 0.001
Forns, Median (IQR)	7.62 (6.52, 9.13)	7.05 (6.11, 8.08)	8.53 (7.09, 10.18)	< 0.001
PRP, Median (IQR)	0.07 (0.06, 0.09)	0.07 (0.06, 0.08)	0.08 (0.06, 0.11)	< 0.001
FIB-5, Mean ± SD	40.2 ± 4.59	40.95 ± 3.84	39.55 ± 5.09	0.019

Abbreviations: CHI3L1: chitinase-3-like protein 1; CHB: chronic hepatitis B; AIH: autoimmune hepatitis; OTHERS: including Liver dysfunction, steatohepatitis, drug-induced hepatitis, and alcoholic hepatitis; AST, Aspartate aminotransferase; ALT, Alanine aminotransferase; GGT, Glutamyl transpeptidase; ALB: Albumin; CHOL: Cholesterol; RDW: Red Cell volume Distribution Width; PLT: Platelet; ALP: Alkaline phosphatase; LSM: liver stiffness; APRI: Aspartate transaminase-to-platelet ratio index; IQR: Interquartile Range.

### 3.2. Six serological models, CHI3L1 and LSM in patients with different stages of liver fibrosis

Based on liver biopsy findings, patients were stratified into five groups according to fibrosis stage: S0 (no fibrosis, n = 36), S1 (mild fibrosis, n = 74), S2 (moderate fibrosis, n = 80), S3 (severe fibrosis, n = 28), and S4 (early cirrhosis, n = 17).

Analysis of non-invasive markers across fibrosis stages revealed distinct patterns (Fig 2). Both FIB-4 and CHI3L1 showed significant differences when comparing S1 with advanced stages (S3 and S4). In contrast, FIB-5, S-Index, and PRP demonstrated limited utility in distinguishing S1 from other advanced fibrosis stages. Notably, APRI, Forns, and LSM exhibited a marked progressive increase across fibrosis stages from S1 to S4. Compared to S1, all subsequent stages (S2, S3, and S4) showed significantly higher values for these three indicators (P < 0.05 for each), suggesting that they may serve as sensitive markers for detecting the progression of liver fibrosis, even at early stages.

### 3.3. Correlations among liver biopsy staging, CHI3L1, LSM, and serological models

Spearman’s correlation analysis was performed to evaluate the relationships among liver biopsy staging, CHI3L1, LSM, and six serological fibrosis models, with the complete correlation matrix presented in Table 2. With the exception of the correlations between Forns and biopsy, Forns and LSM, as well as FIB-5 and biopsy, all other pairwise correlations were statistically significant. Among all parameters, liver biopsy staging showed the strongest correlation with LSM (r = 0.70, p < 0.0001). Notably, FIB-5 exhibited negative correlations with the other serological models, whereas all remaining significant correlations were positive.

**Table 2:** The correlation among biopsy, CHI3L1, LSM, and serological model.

Variable	Biopsy	CHI3L1	LSM	APRI	FIB4	S-Index	Forns	PRP	FIB5
Biopsy	1	0.48*	0.70*	0.3*	0.43*	0.24*	0.47	0.34*	−0.22
CHI3L1	0.48*	1	0.44*	0.26*	0.55*	0.31*	0.52*	0.45*	−0.19*
LSM	0.70*	0.44*	1	0.23*	0.37*	0.30*	0.43	0.33*	−0.24*
APRI	0.30*	0.26*	0.23*	1	0.72*	0.55*	0.52*	0.37*	−0.34*
FIB4	0.43*	0.55*	0.37*	0.72*	1	0.54*	0.75*	0.70*	−0.23*
S-Index	0.24*	0.31*	0.30*	0.55*	0.54*	1	0.60*	0.43*	−0.47*
Forns	0.47	0.52*	0.43	0.52*	0.75*	0.60*	1	0.70*	−0.57*
PRP	0.34*	0.45*	0.33*	0.37*	0.70*	0.43*	0.7*	1	−0.50*
FIB5	−0.22	−0.19*	−0.24*	−0.34*	−0.23*	−0.47*	−0.57*	−0.50*	1

*: Statistically significant, P < 0.05. Abbreviations: CHI3L1, chitinase-3-like protein 1; liver stiffness: LSM; Aspartate transaminase-to-platelet ratio index: APRI.

### 3.4. Performance of CHI3L1, LSM, and serological models in fibrosis stage assessment

LSM demonstrated excellent diagnostic accuracy, with AUROC values of 0.86 for detecting significant fibrosis (≥S2), 0.91 for advanced fibrosis (≥S3), and 0.93 for cirrhosis (S4) (Fig 3). Using the Youden index to determine optimal cut-offs, LSM achieved a sensitivity of 68% and specificity of 86% for ≥S2 at a cut-off of 8.15 kPa, 86% and 82% for ≥S3 at 11.25 kPa, and 80% and 94% for S4 at 13.55 kPa. CHI3L1 showed moderate diagnostic performance, with corresponding AUROC values of 0.72, 0.79, and 0.81. The optimal cut-off values, sensitivity, specificity, PPV, and NPV for LSM, CHI3L1, and all six serological models across the three fibrosis stages are summarized in S1 Table. Among the six serological models, the AUROC values for diagnosing significant fibrosis (≥S2) were as follows: APRI (0.73), FIB‑4 (0.74), S‑Index (0.74), Forns (0.71), PRP (0.65), and FIB‑5 (0.59). For advanced fibrosis (≥S3), the AUROC values were: APRI (0.75), FIB‑4 (0.78), S‑Index (0.73), Forns (0.75), PRP (0.74), and FIB‑5 (0.59). For cirrhosis (S4), the AUROC values were: APRI (0.79), FIB‑4 (0.85), S‑Index (0.78), Forns (0.86), PRP (0.92), and FIB‑5 (0.72) (Fig 3).

**Fig 3:**
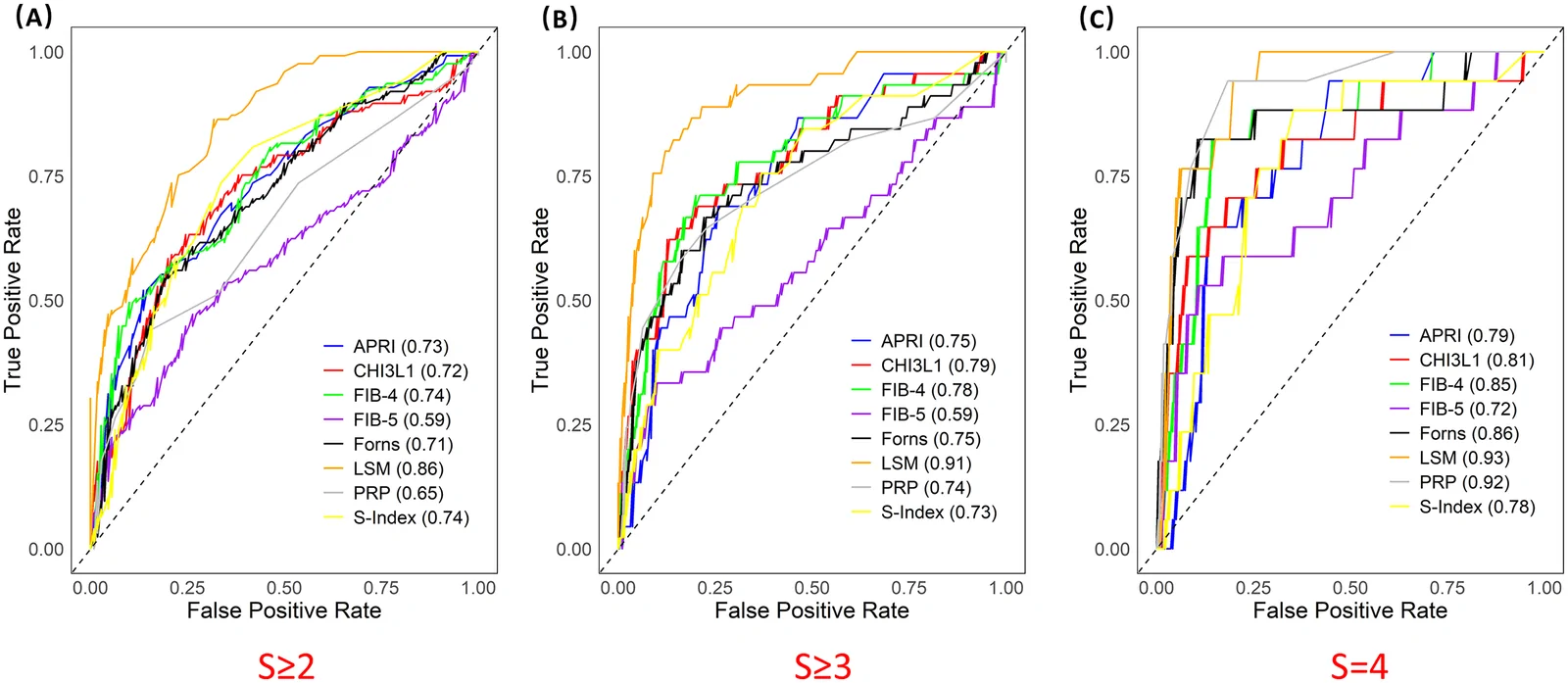
AUROCs for predicting significant fibrosis (≥S2), advanced fibrosis (≥S3), and cirrhosis (S4) among chronic liver diseases. AUROC, The area under the receiver operating characteristic curve; CHI3L1, Chitinase-3-like protein 1; APRI, Aspartate aminotransferase-to-platelet ratio index.

We further performed pairwise comparisons of AUROCs using DeLong’s test to determine whether the observed differences were statistically significant. As shown in S2 Table, LSM exhibited significantly higher AUROC values compared to CHI3L1 and all six serological models for significant fibrosis (≥S2) and advanced fibrosis (≥S3) (all P < 0.05). For cirrhosis (S4), LSM also showed significantly higher AUROCs than APRI, S-Index, and FIB-5 (all P < 0.05). Interestingly, while LSM (AUROC = 0.93) numerically outperformed CHI3L1, FIB-4, Forns, and PRP, the DeLong’s test indicated that this specific difference did not reach statistical significance. Overall, LSM exhibited good diagnostic performance compared to CHI3L1 and all six serological models.

## 4. Discussion

In this real-world, biopsy-proven cohort of patients with CLD, we found that LSM outperformed both CHI3L1 and all six serological models in diagnosing liver fibrosis across all stages. The superior performance of LSM likely stems from its direct measurement of liver tissue stiffness, a physical property intrinsically linked to the amount and architecture of fibrotic tissue [22,23].

In contrast, CHI3L1, while reflecting fibrogenesis and inflammation, is an indirect serum marker influenced by systemic factors and extra-hepatic sources [13,24]. The results demonstrated that levels of LSM, CHI3L1, and the serological models were significantly elevated in patients with liver fibrosis compared to those without. Furthermore, LSM values increased progressively with fibrosis severity. Correlation analysis revealed that liver biopsy staging was positively correlated with LSM, CHI3L1, APRI, FIB‑4, S‑Index, Forns, and PRP.

Numerous studies have investigated the diagnostic value of CHI3L1 in various chronic liver diseases, demonstrating promising performance in chronic hepatitis B (CHB), non‑alcoholic fatty liver disease (NAFLD), and autoimmune hepatitis (AIH). However, several limitations should be noted. One study [25] reported that CHI3L1 achieved a higher AUROC than LSM; however, its small sample size (50 biopsy‑proven patients) limits the generalizability of the findings. Another study involving 78 biopsy‑proven CHB patients [26] reported an AUROC of 0.818 for CHI3L1, but LSM was not performed for comparison. A further study [27] that used LSM as the reference standard in 134 CHB patients found that CHI3L1 yielded an AUROC of only 0.728 for detecting significant fibrosis. Studies evaluating CHI3L1 in NAFLD or AIH populations have also been constrained by small sample sizes [16] or a lack of direct comparison with LSM [13,28]. In contrast, the present study included 235 patients with CLD who underwent liver biopsy, LSM, CHI3L1 testing, and comprehensive laboratory assessments. Our real‑world results demonstrated that CHI3L1 was inferior to LSM in diagnostic performance for liver fibrosis.

From a health economics perspective, the superiority of LSM becomes even more compelling. As China transitions into an aging society, the implementation of Diagnosis‑Related Groups (DRG) and Diagnosis‑Intervention Packet (DIP) payment systems requires clinicians to achieve maximum medical benefit with minimal economic investment. Notably, the cost of CHI3L1 testing is substantially higher than that of LSM. According to the current healthcare fee schedule in Hangzhou, Zhejiang Province, a single CHI3L1 serum assay costs 380 RMB, whereas a FibroTouch examination costs only 88 RMB, making CHI3L1 approximately 4.3 times more expensive than LSM. This translates to a direct cost saving of nearly 300 RMB per patient when LSM is used instead of CHI3L1. Given the substantial healthcare burden in China, LSM offers a practical advantage as a routine tool for monitoring liver fibrosis in patients with CLD, owing to its superior diagnostic performance and lower cost. Widespread adoption of LSM for routine monitoring could therefore contribute to more efficient resource allocation within the DRG/DIP framework by potentially reducing the need for more expensive or invasive procedures. Nevertheless, some studies have suggested that CHI3L1 may provide additional diagnostic value in specific CLD etiologies, such as AIH [13]. Therefore, we propose a risk‑stratified approach: for patients with uncomplicated CLD (e.g., chronic hepatitis B or metabolic-associated steatotic liver disease), LSM alone is recommended as the primary modality for surveillance and follow‑up; for those with favorable economic status and suspected or confirmed AIH, a combination of LSM and CHI3L1 may be considered to optimize diagnostic accuracy.

This study has several limitations. First, the single‑center and retrospective design may introduce selection bias, as our cohort was recruited from a tertiary referral hospital, which likely overrepresents patients with advanced fibrosis and underrepresents those with mild disease. This could inflate the diagnostic performance estimates (e.g., AUROC, sensitivity, and specificity) and limit the generalizability of our findings to community‑based or primary care settings, where the prevalence of significant fibrosis is lower. Therefore, the optimal cut‑off values derived from this cohort should be validated in independent, prospective, multicenter studies encompassing a broader spectrum of CLD etiologies and disease severities before widespread clinical application. It should be acknowledged that the high proportion of advanced fibrosis in our cohort could lead to an overestimation of positive predictive value (PPV) and an underestimation of negative predictive value (NPV) if these results are extended to low-prevalence general screening settings. As such, our findings are best positioned for use in secondary and tertiary hepatology practices, while extrapolation to primary care should be undertaken with due caution. Moreover, prospective validation in community-based cohorts remains essential to confirm the generalizability of these findings. Second, potential confounders, such as age, sex, hepatic inflammation, cholestasis, food intake and body mass index may affect the accuracy of LSM and CHI3L1 measurements. Consequently, the observed diagnostic performance may not be fully attributable to fibrosis alone. Clinicians should interpret the results within the broader clinical context, and future prospective studies should incorporate these variables into multivariable models to refine diagnostic algorithms. Third, although this is the largest biopsy proven cohort, the sample size (n = 235) is modest. Declining use of liver biopsies constrains larger histology confirmed cohorts, and the lack of external validation prevents reproducibility assessment. Future multicenter studies are planned to validate our findings. Finally, we acknowledge that a formal cost effectiveness analysis incorporating quality adjusted life years (QALYs) or incremental cost effectiveness ratios (ICERs) is beyond the scope of this retrospective diagnostic study. Future prospective health economic evaluations are warranted to fully validate the cost benefit profile of LSM versus CHI3L1 across different CLD populations and healthcare settings.

In this biopsy-proven cohort of Chinese patients with CLD, LSM demonstrated superior diagnostic performance for liver fibrosis assessment compared to CHI3L1 and six serological models, exhibiting the strongest correlation with histological staging and the highest AUROC values across all fibrosis stages. Given its better diagnostic efficacy, lower cost, and the constraints of the DRG/DIP payment systems in China, LSM offers a practical and cost-effective advantage as a first-line routine monitoring tool. CHI3L1 may serve as a complementary marker in selected cases, such as patients with suspected AIH, where it may provide additional etiological insight.

## Supporting information

S1 Table Diagnostic performance of LSM, CHI3L1, and serological models for detecting liver fibrosis stages. (DOCX)

S2 Table Pairwise comparison of AUROCs between LSM and other non-invasive markers using DeLong’s test. (DOCX)

S1 File The original data and code used in this study. (ZIP)
