# Supplementary material for: Superior diagnostic efficacy and cost advantage of FibroTouch over CHI3L1 for liver fibrosis in Chinese patients with chronic liver disease
Source: PLoS One. 2026 Oct 5;21(10):e0357883. doi: 10.1371/journal.pone.0357883 (PMC13637865; doi:10.1371/journal.pone.0357883)
Supplement: Supplementary file 1 [file pone.0357883.s004.pdf]

This manuscript focuses on comparing the diagnostic performance and economic benefits between FibroTouch and CHI3L1 for liver fibrosis assessment in Chinese patients with chronic liver disease. The research topic is clinically meaningful, and the sample size is acceptable with liver biopsy as the gold standard. However, multiple major flaws exist in study design, statistical analysis, data consistency, experimental quality control, presentation of results and manuscript writing. Therefore, a Major Revision is required. The detailed comments are listed as follows:

1. Patients undergoing liver biopsy often have more severe or "suspicious" symptoms, which might lead to an overrepresentation of advanced fibrosis (53.2% ≥S2). Please discuss how this prevalence affects the predictive values compared to a general screening population.
2. The manuscript states 235 enrolled patients in the main text, while the ROC curve legend marks the AUC value of LSM as 0.856 for significant fibrosis (≥S2), which conflicts with the text result of 0.850. All inconsistent numerical results need unified revision and verification.
3. The study adopted multiple comparison analyses among different fibrosis stages, but it only mentions using One-Way ANOVA and Dunn's test in figure legends. The detailed application conditions and statistical results of multiple comparison tests are not supplemented in the Methods or Results section.
4. The authors conclude that FibroTouch (LSM) is superior to CHI3L1 and serological models based on AUROC values, but no pairwise comparison of ROC curves (such as DeLong's test) was performed. It is impossible to confirm whether the differences in diagnostic efficacy are statistically significant.
5. While AUROC values are provided, the optimal cut-off values, sensitivity, specificity, positive predictive value (PPV), and negative predictive value (NPV) for each stage (≥S2, ≥S3, S4) are missing. These parameters are crucial for clinical decision-making. Please add a summary table for these diagnostic metrics.
6. The authors briefly mention single-center and retrospective limitations, but fail to further analyze the influence of these limitations on clinical application and result extrapolation. Meanwhile, potential confounding factors affecting CHI3L1 and LSM detection results are not discussed.
7. There are multiple grammatical and spelling errors in the full text. For example, "no competing interests exits" should be revised to "no competing interests exist"; the expression "Diagnostic of FibroTouch" in references is grammatically wrong. In addition, abbreviations are not standardized in some places.
8. The manuscript claims that LSM has an obvious cost advantage over CHI3L1, but only gives a qualitative description of "more than twice the cost". The specific charging standard, medical economic analysis and applicable scenarios are not supplemented.
9. Figure 2 has unclear label marking and incomplete group annotation; Table 1 arranges multiple indicators in a compact layout, and the grouping logic of partial data is confusing. The authors need to optimize the layout and supplement detailed legends.
10. Why were these specific six models (especially FIB-5 and PRP) chosen as comparators? Some, like FIB-5, showed very poor performance (AUROC < 0.6). It would be helpful to justify their relevance or compare with more widely accepted tools like the Enhanced Liver Fibrosis (ELF) score if available.
