# Supplementary material for: Superior diagnostic efficacy and cost advantage of FibroTouch over CHI3L1 for liver fibrosis in Chinese patients with chronic liver disease
Source: PLoS One. 2026 Oct 5;21(10):e0357883. doi: 10.1371/journal.pone.0357883 (PMC13637865; doi:10.1371/journal.pone.0357883)
Supplement: Supplementary file 2 [file pone.0357883.s001.docx]

Supplementary Table S1. Diagnostic performance of LSM, CHI3L1, and serological models for detecting liver fibrosis stages

|  | Significant fibrosis (≥S2) | | | | | Advanced fibrosis (≥S3) | | | | | Cirrhosis (S4) | | | | |
| --- | --- | --- | --- | --- | --- | --- | --- | --- | --- | --- | --- | --- | --- | --- | --- |
| Variable | cut-off | Se | Sp | PPV | NPV | cut-off | Se | Sp | PPV | NPV | cut-off | Se | Sp | PPV | NPV |
| CHI3L1 | 60.98 | 0.75 | 0.63 | 0.64 | 0.75 | 126.89 | 0.87 | 0.62 | 0.91 | 0.54 | 127.78 | 0.82 | 0.71 | 0.97 | 0.24 |
| LSM | 8.15 | 0.68 | 0.86 | 0.82 | 0.76 | 11.25 | 0.86 | 0.82 | 0.95 | 0.59 | 13.55 | 0.80 | 0.94 | 0.99 | 0.27 |
| APRI | 0.68 | 0.86 | 0.52 | 0.61 | 0.81 | 0.69 | 0.74 | 0.69 | 0.91 | 0.39 | 1.46 | 0.87 | 0.65 | 0.97 | 0.28 |
| FIB4 | 2.13 | 0.90 | 0.50 | 0.61 | 0.85 | 2.15 | 0.80 | 0.71 | 0.92 | 0.48 | 3.28 | 0.86 | 0.82 | 0.98 | 0.31 |
| S-Index | 0.095 | 0.66 | 0.74 | 0.69 | 0.71 | 0.15 | 0.63 | 0.76 | 0.92 | 0.33 | 0.20 | 0.65 | 0.88 | 0.98 | 0.16 |
| Forns | 8.31 | 0.81 | 0.54 | 0.61 | 0.76 | 8.72 | 0.78 | 0.67 | 0.91 | 0.42 | 10.17 | 0.89 | 0.82 | 0.98 | 0.38 |
| PRP | 0.085 | 0.85 | 0.44 | 0.57 | 0.76 | 0.095 | 0.84 | 0.58 | 0.89 | 0.46 | 1.12 | 0.82 | 0.94 | 0.99 | 0.29 |
| FIB5 | 38.98 | 0.26 | 0.53 | 0.33 | 0.45 | 41.39 | 0.33 | 0.56 | 0.44 | 0.51 | 43.59 | 0.10 | 0.47 | 0.72 | 0.14 |

Abbreviations: LSM, liver stiffness measurement; CHI3L1, chitinase-3-like protein 1; APRI, aspartate aminotransferase-to-platelet ratio index; PPV, positive predictive value; NPV, negative predictive value; Se: Sensitivity; Sp: Specificity.
