# Supplementary material for: Superior diagnostic efficacy and cost advantage of FibroTouch over CHI3L1 for liver fibrosis in Chinese patients with chronic liver disease
Source: PLoS One. 2026 Oct 5;21(10):e0357883. doi: 10.1371/journal.pone.0357883 (PMC13637865; doi:10.1371/journal.pone.0357883)
Supplement: Supplementary file 3 [file pone.0357883.s002.docx]

Supplementary Table S2. Pairwise comparison of AUROCs between LSM and other non-invasive markers using DeLong’s test.

| Marker | Significant fibrosis (≥S2) | Advanced fibrosis (≥S3) | Cirrhosis (S4) |
| --- | --- | --- | --- |
| LSM vs. CHI3L1 | **<0.001** | **0.004** | 0.08 |
| LSM vs. APRI | **0.001** | **<0.001** | **0.002** |
| LSM vs. FIB-4 | **0.001** | **0.003** | 0.13 |
| LSM vs. S-Index | **0.002** | **<0.001** | **0.006** |
| LSM vs. Forns | **<0.001** | **<0.001** | 0.30 |
| LSM vs. PRP | **<0.001** | **<0.001** | 0.88 |
| LSM vs. FIB-5 | **<0.001** | **<0.001** | **0.003** |

Abbreviations: LSM, liver stiffness measurement; CHI3L1, chitinase-3-like protein 1; APRI, aspartate aminotransferase-to-platelet ratio index. Notes: Values are two-sided p-values from DeLong’s test for paired ROC curves. Statistically significant differences (p < 0.05) are highlighted.
