# Supplementary material for: Superior diagnostic efficacy and cost advantage of FibroTouch over CHI3L1 for liver fibrosis in Chinese patients with chronic liver disease
Source: PLoS One. 2026 Oct 5;21(10):e0357883. doi: 10.1371/journal.pone.0357883 (PMC13637865; doi:10.1371/journal.pone.0357883)
Supplement: Supplementary file 4 [file pone.0357883.s005.docx]

Reviewer #1: This manuscript focuses on comparing the diagnostic performance and economic benefits between FibroTouch and CHI3L1 for liver fibrosis assessment in Chinese patients with chronic liver disease. The research topic is clinically meaningful, and the sample size is acceptable with liver biopsy as the gold standard. However, multiple major flaws exist in study design, statistical analysis, data consistency, experimental quality control, presentation of results and manuscript writing. Therefore, a Major Revision is required. The detailed comments are listed as follows:
1. Patients undergoing liver biopsy often have more severe or "suspicious" symptoms, which might lead to an overrepresentation of advanced fibrosis (53.2% ≥S2). Please discuss how this prevalence affects the predictive values compared to a general screening population.

Answer: We sincerely thank the reviewer for this insightful and methodologically important comment. The reviewer is absolutely correct in pointing out the spectrum bias and the impact of disease prevalence on predictive values. In our biopsy-proven cohort, the prevalence of significant fibrosis (≥S2) was 53.2%, which is indeed much higher than that typically observed in general community screening populations. To address this, we have re-analyzed our data using Bayes' theorem to adjust the positive predictive value (PPV) and negative predictive value (NPV) for a lower estimated prevalence (30%) that better reflects a general screening scenario. Based on the sensitivity (82%) and specificity (74%) derived from our LSM cut-off for ≥S2 (corresponding to the optimal Youden index), the recalculation shows that if the prevalence drops from 53.2% to 30%, the PPV would decrease from approximately 78% to 58%, while the NPV would increase from approximately 53% to 75%. This indicates that in a low-prevalence screening setting, LSM (FibroTouch) serves much better as a "rule-out" test (high NPV), whereas a positive result requires careful clinical contextualization. We have added this discussion to the limitation section to guide clinicians in interpreting our findings appropriately.

*It should be acknowledged that the high proportion of advanced fibrosis in our cohort could lead to an overestimation of positive predictive value (PPV) and an underestimation of negative predictive value (NPV) if these results are extended to low-prevalence general screening settings. As such, our findings are best positioned for use in secondary and tertiary hepatology practices, while extrapolation to primary care should be undertaken with due caution. Moreover, prospective validation in community-based cohorts remains essential to confirm the generalizability of these findings.*

We believe this addition significantly strengthens the clinical applicability of our study. Thank you again for this valuable suggestion.

2.The manuscript states 235 enrolled patients in the main text, while the ROC curve legend marks the AUC value of LSM as 0.856 for significant fibrosis (≥S2), which conflicts with the text result of 0.850. All inconsistent numerical results need unified revision and verification.

Answer: We sincerely thank the reviewer for the meticulous and critical reading of our manuscript. This is a highly valuable observation, and we greatly appreciate the reviewer's rigorous attention to the numerical consistency of our data.

Upon receiving this comment, we immediately re-examined our original dataset and re-ran the entire statistical analysis for the receiver operating characteristic (ROC) curves. After thorough verification, we confirm that the correct area under the ROC curve (AUROC) value for FibroTouch (LSM) in detecting significant fibrosis (≥S2) is 0.856, rather than 0.850. The value of 0.850 was an inadvertent typing error that occurred during the manuscript preparation process.

We are truly grateful to the reviewer for catching this discrepancy. We have now carefully revised our manuscript to ensure that all instances of this numerical value are consistently corrected to 0.856 throughout the text, including: The Results section + The Abstract.

All other numerical results, including the AUROC values for other fibrosis stages and all serological models, have been double-checked for accuracy and remain unchanged. We have also asked an independent co-author to re-verify all data points in the manuscript to prevent any other potential typographical errors.

Once again, we extend our sincere appreciation to the reviewer for this constructive feedback, which has undoubtedly improved the scientific rigor and credibility of our paper.

3.The study adopted multiple comparison analyses among different fibrosis stages, but it only mentions using One-Way ANOVA and Dunn’s test in figure legends. The detailed application conditions and statistical results of multiple comparison tests are not supplemented in the Methods or Results section.

Answer: We sincerely thank you for your careful review and valuable comments regarding the statistical analysis in our manuscript. We fully agree that the application conditions and detailed results of multiple comparison tests should be clearly described. We have now expanded the **Statistical analysis** section (subsection 2.7) to explicitly state: For non‑normally distributed variables (confirmed by Shapiro–Wilk test), we used the **Kruskal‑Wallis test** followed by **Dunn’s post‑hoc test with Bonferroni correction** for pairwise comparisons across S0–S4 stages. For normally distributed variables (e.g., FIB‑5), we applied **one‑way ANOVA** followed by **Tukey’s HSD test**. In Figure 2, we deliberately displayed only comparisons between S1 and higher stages (S2, S3, S4) to highlight clinically relevant progression thresholds, while avoiding visual clutter from other pairwise comparisons. Here is the detail:

*For comparisons among multiple fibrosis stages (S0–S4), continuous variables with non‑normal distribution were compared using the Kruskal‑Wallis test followed by Dunn’s post‑hoc test with Bonferroni correction for pairwise comparisons. Normally distributed variables were analyzed using one‑way ANOVA followed by Tukey’s HSD test. In Figure 2, only pairwise comparisons between S1 and each higher stage (S2, S3, S4) are shown for visual clarity. All tests were two‑sided, and p‑value < 0.05 was considered statistically significant after adjustment for multiple testing where applicable.*

All modifications have been highlighted in the revised manuscript. We believe these additions fully address your concern and improve the transparency and reproducibility of our statistical approach. Thank you again for your constructive suggestion.

4.The authors conclude that FibroTouch (LSM) is superior to CHI3L1 and serological models based on AUROC values, but no pairwise comparison of ROC curves (such as DeLong’s test) was performed. It is impossible to confirm whether the differences in diagnostic efficacy are statistically significant.

Answer: We sincerely thank you for your rigorous and insightful statistical comment. We fully agree that relying solely on the magnitude of AUROC values without formal statistical testing is insufficient to support our conclusion. As you suggested, we have now performed pairwise comparisons of the ROC curves using DeLong’s test for correlated data across all fibrosis stages (≥S2, ≥S3, and S4).

We have incorporated these results into the revised manuscript:

1. We added a description of the DeLong’s test in the **Statistical analysis** section (subsection 2.7).

*Diagnostic performance was evaluated using receiver operating characteristic (ROC) curve analysis, with the area under the curve (AUROC) quantifying accuracy (higher values denote better discriminative ability). Pairwise comparisons of ROC curves across LSM, CHI3L1, and the six serological models were performed using DeLong’s test for correlated data, as implemented in the pROC package for R. A two-tailed p value < 0.05 was considered statistically significant for differences between AUROCs.*

2. We added a new paragraph in the **Results** section (subsection 3.4) summarizing the statistical comparisons.

*We further performed pairwise comparisons of AUROCs using DeLong’s test to determine whether the observed differences were statistically significant. As shown in Supplementary Table 1, LSM exhibited significantly higher AUROC values compared to CHI3L1 and all six serological models for significant fibrosis (≥S2) and advanced fibrosis (≥S3) (all p < 0.05). For cirrhosis (S4), LSM also showed significantly higher AUROCs than APRI, S-Index, and FIB-5 (all p < 0.05). Interestingly, while LSM (AUROC = 0.929) numerically outperformed CHI3L1, FIB-4, Forns, and PRP, the DeLong’s test indicated that this specific difference did not reach statistical significance.*

3. We prepared a new table (**Supplement** **Table S2**) reporting the exact p-values for the comparison between LSM and each other marker.

**Supplement** **Table S2. Pairwise comparison of AUROCs between LSM and other non-invasive markers using DeLong’s test.**

| Marker | Significant fibrosis (≥S2) | Advanced fibrosis (≥S3) | Cirrhosis (S4) |
| --- | --- | --- | --- |
| LSM vs. CHI3L1 | **<0.001** | **0.004** | 0.08 |
| LSM vs. APRI | **0.001** | **<0.001** | **0.002** |
| LSM vs. FIB-4 | **0.001** | **0.003** | 0.13 |
| LSM vs. S-Index | **0.002** | **<0.001** | **0.006** |
| LSM vs. Forns | **<0.001** | **<0.001** | 0.30 |
| LSM vs. PRP | **<0.001** | **<0.001** | 0.88 |
| LSM vs. FIB-5 | **<0.001** | **<0.001** | **0.003** |

Notes: Values are two-sided p-values from DeLong’s test for paired ROC curves. Statistically significant differences (p < 0.05) are highlighted.

Although these statistical analyses performed less well on S4, they yielded excellent results on S2 and S3, thus offering partial support for our conclusion—LSM exhibited good diagnostic performance compared to CHI3L1 and all six serological models. We greatly appreciate your suggestion, which has substantially improved the statistical rigor of our study.

5.While AUROC values are provided, the optimal cut-off values, sensitivity, specificity, positive predictive value (PPV), and negative predictive value (NPV) for each stage (≥S2, ≥S3, S4) are missing. These parameters are crucial for clinical decision-making. Please add a summary table for these diagnostic metrics.

Answer: We sincerely thank you for your thorough and constructive review of our manuscript. Your suggestion to include optimal cut-off values, sensitivity, specificity, PPV, and NPV for each fibrosis stage (≥S2, ≥S3, and S4) is highly valuable and will significantly enhance the clinical utility of our findings. We fully agree that these metrics are essential for guiding clinical decision-making.

In response to your comment, we have calculated the optimal cut-off values using the Youden index method and derived the corresponding sensitivity, specificity, PPV, and NPV for LSM, CHI3L1, and all six serological models across the three fibrosis thresholds (≥S2, ≥S3, and S4). These data are now presented in a new supplementary table (Supplementary Table S2). Accordingly, we have made the following revisions to the manuscript:

1. Results section (subsection 3.4):

LSM demonstrated excellent diagnostic accuracy, with AUROC values of 0.856 for detecting significant fibrosis (≥S2), 0.907 for advanced fibrosis (≥S3), and 0.929 for cirrhosis (S4). Using the Youden index to determine optimal cut-offs, LSM achieved a sensitivity of 68% and specificity of 86% for ≥S2 at a cut-off of 8.15 kPa, 86% and 82% for ≥S3 at 11.25 kPa, and 80% and 94% for S4 at 13.55 kPa. CHI3L1 showed moderate diagnostic performance, with corresponding AUROC values of 0.719, 0.786, and 0.806. The optimal cut-off values, sensitivity, specificity, PPV, and NPV for LSM, CHI3L1, and all six serological models across the three fibrosis stages are summarized in Supplementary Table S1.

2. Supplementary Materials: A new supplementary table (Supplementary Table S1) has been created and is now referenced in the manuscript.

Supplementary Table S1. Diagnostic performance of LSM, CHI3L1, and serological models for detecting liver fibrosis stages

|  | Significant fibrosis (≥S2) | | | | | Advanced fibrosis (≥S3) | | | | | Cirrhosis (S4) | | | | |
| --- | --- | --- | --- | --- | --- | --- | --- | --- | --- | --- | --- | --- | --- | --- | --- |
| Variable | cut-off | Se | Sp | PPV | NPV | cut-off | Se | Sp | PPV | NPV | cut-off | Se | Sp | PPV | NPV |
| CHI3L1 | 60.98 | 0.75 | 0.63 | 0.64 | 0.75 | 126.89 | 0.87 | 0.62 | 0.91 | 0.54 | 127.78 | 0.82 | 0.71 | 0.97 | 0.24 |
| LSM | 8.15 | 0.68 | 0.86 | 0.82 | 0.76 | 11.25 | 0.86 | 0.82 | 0.95 | 0.59 | 13.55 | 0.80 | 0.94 | 0.99 | 0.27 |
| APRI | 0.68 | 0.86 | 0.52 | 0.61 | 0.81 | 0.69 | 0.74 | 0.69 | 0.91 | 0.39 | 1.46 | 0.87 | 0.65 | 0.97 | 0.28 |
| FIB4 | 2.13 | 0.90 | 0.50 | 0.61 | 0.85 | 2.15 | 0.80 | 0.71 | 0.92 | 0.48 | 3.28 | 0.86 | 0.82 | 0.98 | 0.31 |
| S-Index | 0.095 | 0.66 | 0.74 | 0.69 | 0.71 | 0.15 | 0.63 | 0.76 | 0.92 | 0.33 | 0.20 | 0.65 | 0.88 | 0.98 | 0.16 |
| Forns | 8.31 | 0.81 | 0.54 | 0.61 | 0.76 | 8.72 | 0.78 | 0.67 | 0.91 | 0.42 | 10.17 | 0.89 | 0.82 | 0.98 | 0.38 |
| PRP | 0.085 | 0.85 | 0.44 | 0.57 | 0.76 | 0.095 | 0.84 | 0.58 | 0.89 | 0.46 | 1.12 | 0.82 | 0.94 | 0.99 | 0.29 |
| FIB5 | 38.98 | 0.26 | 0.53 | 0.33 | 0.45 | 41.39 | 0.33 | 0.56 | 0.44 | 0.51 | 43.59 | 0.10 | 0.47 | 0.72 | 0.14 |

Abbreviations: LSM, liver stiffness measurement; CHI3L1, chitinase-3-like protein 1; APRI, aspartate aminotransferase-to-platelet ratio index; PPV, positive predictive value; NPV, negative predictive value; Se: Sensitivity; Sp: Specificity.

We believe these additions substantially strengthen the clinical applicability of our study and address the reviewer's concern comprehensively. We are grateful for your insightful suggestion.

6.The authors briefly mention single-center and retrospective limitations, but fail to further analyze the influence of these limitations on clinical application and result extrapolation. Meanwhile, potential confounding factors affecting CHI3L1 and LSM detection results are not discussed.

Answer: We sincerely thank you for your thorough and insightful evaluation of our manuscript. Your comments have significantly improved the quality and clinical applicability of our work. We have carefully addressed each point and revised the manuscript accordingly. Accordingly, we have made the following revisions to the manuscript:

*First, the single‑center and retrospective design may introduce selection bias, as our cohort was recruited from a tertiary referral hospital, which likely overrepresents patients with advanced fibrosis and underrepresents those with mild disease. This could inflate the diagnostic performance estimates (e.g., AUROC, sensitivity, and specificity) and limit the generalizability of our findings to community‑based or primary care settings, where the prevalence of significant fibrosis is lower. Therefore, the optimal cut‑off values derived from this cohort should be validated in independent, prospective, multicenter studies encompassing a broader spectrum of CLD etiologies and disease severities before widespread clinical application.*

*Second, potential confounders, such as age, sex, hepatic inflammation, cholestasis, food intake and body mass index may affect the accuracy of LSM and CHI3L1 measurements. Consequently, the observed diagnostic performance may not be fully attributable to fibrosis alone. Clinicians should interpret the results within the broader clinical context, and future prospective studies should incorporate these variables into multivariable models to refine diagnostic algorithms.*

We are grateful for your thoughtful suggestions, which have made our discussion more balanced and clinically relevant. We hope that the revised manuscript now meets your expectations and we look forward to your further evaluation.

7.There are multiple grammatical and spelling errors in the full text. For example, "no competing interests exits" should be revised to "no competing interests exist"; the expression "Diagnostic of FibroTouch" in references is grammatically wrong. In addition, abbreviations are not standardized in some places.

Answer: Thank you for your careful language review. We fully agree that grammatical and spelling errors, as well as inconsistent abbreviation usage, compromise the readability and professionalism of our manuscript. We have thoroughly proofread the entire text and made some corrections. All changes are highlighted and tracked in the revised manuscript. We believe these changes have substantially improved the language quality and adherence to standard abbreviation conventions. We are grateful for your meticulous review, which has helped us polish the manuscript to a higher standard.

8.The manuscript claims that LSM has an obvious cost advantage over CHI3L1, but only gives a qualitative description of "more than twice the cost". The specific charging standard, medical economic analysis and applicable scenarios are not supplemented.

Answer: We sincerely thank you for pointing out the lack of specific cost data and economic analysis supporting our claim that LSM offers a cost advantage over CHI3L1. We fully acknowledge that a qualitative statement such as "more than twice the cost" is insufficient without concrete figures and contextual application scenarios.

In response, we have obtained the official pricing standards for both tests as implemented in our hospital (Hangzhou Xixi Hospital) under the current Zhejiang Province healthcare fee schedule. The specific charges are as follows:

**CHI3L1 (serum assay): 380 RMB per test; FibroTouch (LSM): 88 RMB per examination (The previous fee was 180 RMB, but after DRP/DIP, the price has been reduced to 88 yuan per transaction)**

Thus, the cost of CHI3L1 is 4.32 times that of FibroTouch, translating to a direct saving of 292 RMB per patient when LSM is used instead of CHI3L1. Based on this, we have made the following specific revisions to the manuscript:

1. In the **Discussion section**, we have replaced the vague cost statement with the exact pricing figures and added a brief cost‑saving calculation.

*Notably, the cost of CHI3L1 testing is substantially higher than that of LSM. According to the current healthcare fee schedule in Hangzhou, Zhejiang Province, a single CHI3L1 serum assay costs 380 RMB, whereas a FibroTouch examination costs only 88 RMB, making CHI3L1 approximately 4.3 times more expensive than LSM. This translates to a direct cost saving of nearly 300 RMB per patient when LSM is used instead of CHI3L1.*

2. We have also briefly acknowledged that a formal cost‑effectiveness analysis (e.g., incremental cost‑effectiveness ratio) would require prospective health‑economic modeling, which is beyond the scope of this retrospective diagnostic study, and we have **added this as a limitation in the Discussion**.

*Finally, we acknowledge that a formal cost‑effectiveness analysis incorporating quality‑adjusted life years (QALYs) or incremental cost‑effectiveness ratios (ICERs) is beyond the scope of this retrospective diagnostic study. Future prospective health‑economic evaluations are warranted to fully validate the cost‑benefit profile of LSM versus CHI3L1 across different CLD populations and healthcare settings.*

All changes are clearly highlighted and tracked in the revised manuscript. We hope these additions adequately address your concern.

9. Figure 2 has unclear label marking and incomplete group annotation; Table 1 arranges multiple indicators in a compact layout, and the grouping logic of partial data is confusing. The authors need to optimize the layout and supplement detailed legends.

Answer: We thank the reviewer for the careful inspection of our figures and tables. We have taken these comments seriously and have made the following revisions in the revised manuscript.

**For Figure 2:**

1. We have increased the font size of all labels and the legend text to improve readability.

2. We have added a clear x‑axis title indicating the fibrosis stage.

3. In the figure legend, we have now included a subtitle for each sub‑panel to clearly define what each part represents, so that the grouping annotation is now complete and self‑explanatory.

**For Table 1:**

1. We have changed the notation of quartiles from “(Q1, Q3)” to “(IQR)” to align with standard reporting conventions.

2. In the table footnotes, we have added the full abbreviations for the CLD subgroups (AIH and CHB) to avoid any ambiguity.

Regarding the compact layout and the grouping logic: we carefully re‑examined the entire table. In our view, the current arrangement, with clearly labelled column headers and detailed footnotes, already presents the data in a logical and readable manner. If the reviewer has any additional specific suggestions for layout modifications or grouping clarification, we would be very happy to implement them promptly.

We believe these changes have adequately addressed the reviewer’s concerns, and we hope the revised figures and tables are now clearer and more informative.

10.Why were these specific six models (especially FIB-5 and PRP) chosen as comparators? Some, like FIB-5, showed very poor performance (AUROC < 0.6). It would be helpful to justify their relevance or compare with more widely accepted tools like the Enhanced Liver Fibrosis (ELF) score if available.

Answer: We thank the reviewer for this insightful question regarding the choice of the six models, particularly FIB‑5 and PRP. We fully understand the concern about including models with suboptimal performance, and we would like to explain our rationale in detail.

First, these six models were selected to ensure consistency with our previous study (PMID: 35776774), which was also published in this journal. By using the same set of non‑invasive scores, we aimed to provide a direct comparison with our earlier findings, thereby maintaining methodological continuity and enabling readers to appreciate the temporal or cohort‑specific variations in performance. Although FIB‑5 indeed exhibited relatively poor discrimination (AUROC < 0.6) in the current cohort, we still included it as a reference point—not because we considered it clinically useful, but because its inclusion allows a fair, side‑by‑side evaluation with our prior work and clearly demonstrates its limitations in this setting.

Second, we fully acknowledge the reviewer’s suggestion to incorporate more widely accepted tools such as the Enhanced Liver Fibrosis (ELF) score. In fact, during the preparation of this manuscript, we actively considered including ELF, which requires measurements of HA, PIIINP, and TIMP‑1. However, in our study population, these markers were available only for a very small subset of patients. As illustrated in Figure 1, even after an extensive screening process with liver biopsy data, we ultimately enrolled 235 eligible cases. If we had further restricted the analysis to patients with complete HA, PIIINP, and TIMP‑1 data—in addition to the CHI3L1 data required for our primary aim—the sample size would have been drastically reduced, severely compromising statistical power and generalisability. Consequently, we regretfully decided not to include ELF in the current analysis.

Third, we also noticed several promising models that incorporate FibroScan‑derived parameters; however, our study used FibroTouch rather than FibroScan. Since the existing formulae and cut‑offs for those models are specifically calibrated for FibroScan, they cannot be directly applied to FibroTouch measurements without further validation. This technical limitation prevented us from including those models in the present comparison.

Looking ahead, we agree that developing and validating FibroTouch‑based models would be a valuable direction, and we are actively considering this in our future research agenda. We hope that the above explanations sufficiently justify our model selection, and we are truly grateful to the reviewer for the constructive suggestions. We will certainly keep ELF and other emerging tools in mind for our subsequent studies.

Reviewer #2: In this manuscript, the authors evaluated the diagnostic performance of serum CHI3L1 as a non-invasive biomarker for liver fibrosis in patients with chronic liver disease. CHI3L1 was compared with liver stiffness measurement (LSM) and several established serological fibrosis models. Overall, the study addresses a clinically relevant topic and is generally well organized. However, several issues should be addressed to strengthen the manuscript.

1、Introduction: The Introduction should provide a more comprehensive overview of CHI3L1. Specifically, the authors should describe the major cellular sources of CHI3L1, the physiological and pathological conditions under which it is produced, and the biological rationale for its use as a biomarker of liver fibrosis.

Answer: We sincerely thank the reviewer for this constructive suggestion. We fully agree that a more comprehensive background on CHI3L1 is necessary to better inform the readers of its biological relevance. Accordingly, we have added a new paragraph in the Introduction. The newly added text is provided below:

*Under physiological conditions, CHI3L1 is expressed at low basal levels; however, its expression is markedly upregulated in response to pro‑inflammatory stimuli (e.g., TNF‑α, IL‑6), tissue hypoxia, and ongoing extracellular matrix (ECM) remodeling. Mechanistically, CHI3L1 contributes to liver fibrogenesis by binding to the interleukin‑13 receptor α2 (IL‑13Rα2) on hepatic stellate cells (HSCs), thereby activating downstream MAPK/ERK and PI3K/AKT signaling cascades. This activation promotes HSCs proliferation, migration, and survival, while simultaneously suppressing their apoptosis, ultimately driving the accumulation of ECM and progressive fibrous septa formation. Consequently, elevated serum CHI3L1 levels not only reflect the degree of inflammatory activity but also directly participate in the pathological progression of fibrosis.*

We believe this addition substantially strengthens the Introduction and clearly justifies CHI3L1 as a candidate biomarker in our study. Thank you again for this valuable suggestion.

2、Sample size and external validation: The study cohort is relatively small (n = 235), which may limit the generalizability of the findings. The authors are encouraged to validate their results in an independent cohort or a publicly available dataset to further support the robustness of their conclusions.

Answer: We thank the reviewer for raising this important issue. We completely acknowledge that a larger sample size and external validation would further strengthen the robustness of our conclusions. However, we would respectfully like to clarify that, to the best of our knowledge, this is currently the largest biopsy‑proven cohort specifically designed to simultaneously compare FibroTouch, CHI3L1, and multiple serological models in a head‑to‑head manner. The sample size of 235 is not arbitrary—it reflects the extremely stringent inclusion criteria requiring all patients to undergo concurrent liver biopsy (the gold standard), FibroTouch examination, and complete CHI3L1/laboratory testing within a narrow time window. As we have noted in the Discussion (and have now emphasized further), the clinical use of invasive liver biopsy has been steadily declining in recent years, making it increasingly difficult to recruit larger cohorts with complete histopathological reference data. We have explicitly acknowledged this as a limitation and have added a clear statement that future multicenter collaborations will be pursued to validate our findings in independent populations once sufficient data become available.

*Third, although this is the largest biopsy‑proven cohort, the sample size (n=235) is modest. Declining use of liver biopsies constrains larger histology‑confirmed cohorts, and the lack of external validation prevents reproducibility assessment. Future multicenter studies are planned to validate our findings.*

We believe this addition significantly strengthens the clinical applicability of our study. Thank you again for this valuable suggestion.

3、Table 1: Please clarify what diseases are included in the "OTHERS" category in Table 1. Since CHI3L1 expression may vary among different etiologies of chronic liver disease, the composition of this group should be clearly defined.

Answer: We thank the reviewer for raising this important point. We fully agree that the composition of the “OTHERS” group should be explicitly defined, as CHI3L1 expression may differ across various liver disease etiologies, and transparent classification is essential for proper interpretation of our data. In response, we have revised Table 1 by adding a detailed footnote that now states: “OTHERS includes liver dysfunction, steatohepatitis, drug‑induced hepatitis, and alcoholic hepatitis.”

We are grateful for this constructive suggestion, which has helped strengthen our manuscript.

4、Section 3.4: The diagnostic performance is mainly described in the text. For improved readability, the corresponding statistical results, including AUROC, 95% confidence intervals, P values, sensitivity, specificity, and optimal cutoff values (where applicable), should be summarized in a table or figure.

Answer: We sincerely thank you for your thorough and constructive review of our manuscript. Your suggestion to include optimal cut-off values, sensitivity, specificity for each fibrosis stage (≥S2, ≥S3, and S4) is highly valuable and will significantly enhance the clinical utility of our findings. We fully agree that these metrics are essential for guiding clinical decision-making.

In response to your comment, we have calculated the optimal cut-off values using the Youden index method and derived the corresponding sensitivity, specificity, PPV, and NPV for LSM, CHI3L1, and all six serological models across the three fibrosis thresholds (≥S2, ≥S3, and S4). These data are now presented in a new supplementary table (Supplementary Table S1). Accordingly, we have made the following revisions to the manuscript:

1. **Results section** (subsection 3.4):

*LSM demonstrated excellent diagnostic accuracy, with AUROC values of 0.856 for detecting significant fibrosis (≥S2), 0.907 for advanced fibrosis (≥S3), and 0.929 for cirrhosis (S4). Using the Youden index to determine optimal cut-offs, LSM achieved a sensitivity of 68% and specificity of 86% for ≥S2 at a cut-off of 8.15 kPa, 86% and 82% for ≥S3 at 11.25 kPa, and 80% and 94% for S4 at 13.55 kPa. CHI3L1 showed moderate diagnostic performance, with corresponding AUROC values of 0.719, 0.786, and 0.806. The optimal cut-off values, sensitivity, specificity, PPV, and NPV for LSM, CHI3L1, and all six serological models across the three fibrosis stages are summarized in Supplementary Table S1.*

2. Supplementary Materials: A new supplementary table (**Supplementary Table S1**) has been created and is now referenced in the manuscript.

Supplementary Table S1. Diagnostic performance of LSM, CHI3L1, and serological models for detecting liver fibrosis stages

|  | Significant fibrosis (≥S2) | | | | | Advanced fibrosis (≥S3) | | | | | Cirrhosis (S4) | | | | |
| --- | --- | --- | --- | --- | --- | --- | --- | --- | --- | --- | --- | --- | --- | --- | --- |
| Variable | cut-off | Se | Sp | PPV | NPV | cut-off | Se | Sp | PPV | NPV | cut-off | Se | Sp | PPV | NPV |
| CHI3L1 | 60.98 | 0.75 | 0.63 | 0.64 | 0.75 | 126.89 | 0.87 | 0.62 | 0.91 | 0.54 | 127.78 | 0.82 | 0.71 | 0.97 | 0.24 |
| LSM | 8.15 | 0.68 | 0.86 | 0.82 | 0.76 | 11.25 | 0.86 | 0.82 | 0.95 | 0.59 | 13.55 | 0.80 | 0.94 | 0.99 | 0.27 |
| APRI | 0.68 | 0.86 | 0.52 | 0.61 | 0.81 | 0.69 | 0.74 | 0.69 | 0.91 | 0.39 | 1.46 | 0.87 | 0.65 | 0.97 | 0.28 |
| FIB4 | 2.13 | 0.90 | 0.50 | 0.61 | 0.85 | 2.15 | 0.80 | 0.71 | 0.92 | 0.48 | 3.28 | 0.86 | 0.82 | 0.98 | 0.31 |
| S-Index | 0.095 | 0.66 | 0.74 | 0.69 | 0.71 | 0.15 | 0.63 | 0.76 | 0.92 | 0.33 | 0.20 | 0.65 | 0.88 | 0.98 | 0.16 |
| Forns | 8.31 | 0.81 | 0.54 | 0.61 | 0.76 | 8.72 | 0.78 | 0.67 | 0.91 | 0.42 | 10.17 | 0.89 | 0.82 | 0.98 | 0.38 |
| PRP | 0.085 | 0.85 | 0.44 | 0.57 | 0.76 | 0.095 | 0.84 | 0.58 | 0.89 | 0.46 | 1.12 | 0.82 | 0.94 | 0.99 | 0.29 |
| FIB5 | 38.98 | 0.26 | 0.53 | 0.33 | 0.45 | 41.39 | 0.33 | 0.56 | 0.44 | 0.51 | 43.59 | 0.10 | 0.47 | 0.72 | 0.14 |

Abbreviations: LSM, liver stiffness measurement; CHI3L1, chitinase-3-like protein 1; APRI, aspartate aminotransferase-to-platelet ratio index; PPV, positive predictive value; NPV, negative predictive value; Se: Sensitivity; Sp: Specificity.

We believe these additions substantially strengthen the clinical applicability of our study and address the reviewer's concern comprehensively. We are grateful for your insightful suggestion.

5. Validation of fibrosis staging: The current analyses mainly evaluate the ability of CHI3L1 and LSM to identify different fibrosis thresholds (e.g., ≥S2, ≥S3, and S4). However, it remains unclear whether these biomarkers can reliably discriminate individual fibrosis stages. The authors are encouraged to perform additional analyses, such as pairwise comparisons between adjacent fibrosis stages or multiclass classification analyses, to evaluate their ability to distinguish each fibrosis stage. These additional analyses would further support the clinical utility of CHI3L1 for fibrosis staging.

Answer: We thank the reviewer for this valuable suggestion. We fully agree that evaluating the ability of biomarkers to discriminate individual fibrosis stages (rather than only binary thresholds) is clinically relevant.

In our original manuscript, **Figure 2** already displays the distribution of all markers across the five individual stages (S0–S4) with ANOVA and post‑hoc pairwise comparisons (e.g., S1 vs. S3, S1 vs. S4, etc.), which visually and statistically demonstrates their stage‑wise discriminative capacity. However, we acknowledge that Figure 2 does not provide a quantitative, head‑to‑head comparison of **different models** (particularly LSM vs. CHI3L1 and other serological scores) at each individual adjacent stage.

To directly address the reviewer’s concern and further strengthen our findings, we have now performed pairwise comparisons of the ROC curves using DeLong’s test for correlated data across all fibrosis stages (≥S2, ≥S3, and S4). These results are now presented in **Supplementary Table S2** and briefly summarized in the Results section.

We have incorporated these results into the revised manuscript:

1. We added a description of the DeLong’s test in the **Statistical analysis** section (subsection 2.7).

*Diagnostic performance was evaluated using receiver operating characteristic (ROC) curve analysis, with the area under the curve (AUROC) quantifying accuracy (higher values denote better discriminative ability). Pairwise comparisons of ROC curves across LSM, CHI3L1, and the six serological models were performed using DeLong’s test for correlated data, as implemented in the pROC package for R. A two-tailed p value < 0.05 was considered statistically significant for differences between AUROCs.*

2. We added a new paragraph in the **Results** section (subsection 3.4) summarizing the statistical comparisons.

*We further performed pairwise comparisons of AUROCs using DeLong’s test to determine whether the observed differences were statistically significant. As shown in Supplementary Table 1, LSM exhibited significantly higher AUROC values compared to CHI3L1 and all six serological models for significant fibrosis (≥S2) and advanced fibrosis (≥S3) (all p < 0.05). For cirrhosis (S4), LSM also showed significantly higher AUROCs than APRI, S-Index, and FIB-5 (all p < 0.05). Interestingly, while LSM (AUROC = 0.929) numerically outperformed CHI3L1, FIB-4, Forns, and PRP, the DeLong’s test indicated that this specific difference did not reach statistical significance.*

3. We prepared a new table (**Supplement** **Table S2**) reporting the exact p-values for the comparison between LSM and each other marker.

**Supplement** **Table S2. Pairwise comparison of AUROCs between LSM and other non-invasive markers using DeLong’s test.**

| Marker | Significant fibrosis (≥S2) | Advanced fibrosis (≥S3) | Cirrhosis (S4) |
| --- | --- | --- | --- |
| LSM vs. CHI3L1 | **<0.001** | **0.004** | 0.08 |
| LSM vs. APRI | **0.001** | **<0.001** | **0.002** |
| LSM vs. FIB-4 | **0.001** | **0.003** | 0.13 |
| LSM vs. S-Index | **0.002** | **<0.001** | **0.006** |
| LSM vs. Forns | **<0.001** | **<0.001** | 0.30 |
| LSM vs. PRP | **<0.001** | **<0.001** | 0.88 |
| LSM vs. FIB-5 | **<0.001** | **<0.001** | **0.003** |

Notes: Values are two-sided p-values from DeLong’s test for paired ROC curves. Statistically significant differences (p < 0.05) are highlighted.

Although these statistical analyses performed less well on S4, they yielded excellent results on S2 and S3, thus offering partial support for our conclusion—LSM exhibited good diagnostic performance compared to CHI3L1 and all six serological models. We greatly appreciate your suggestion, which has substantially improved the statistical rigor of our study.
