# Supplementary material for: Superior diagnostic efficacy and cost advantage of FibroTouch over CHI3L1 for liver fibrosis in Chinese patients with chronic liver disease
Source: PLoS One. 2026 Oct 5;21(10):e0357883. doi: 10.1371/journal.pone.0357883 (PMC13637865; doi:10.1371/journal.pone.0357883)
Supplement: Supplementary file 5 [file pone.0357883.s006.docx]

Reviewer #2:

1. Figure formatting
Please improve the consistency of figure formatting. For example, in Supplementary Figures S1 and S2, numerical values are reported with inconsistent decimal precision (e.g., some values are presented with two decimal places, whereas others are reported with four decimal places). Please standardize the numerical formatting throughout all figures and tables.
Answer: We thank the reviewer for the careful attention to the formatting details of our figures and tables. We agree that consistency in numerical presentation is important for clarity and have thoroughly revised all figures and tables accordingly.

Specifically, we have made the following changes:

(1). Figures and Tables: We have standardized the numerical formatting throughout all figures and tables ((including Supplementary Table S1 and S2)) to two decimal places for all continuous variables where such precision is appropriate. This includes the AUC values presented in Figure 2, which have now been uniformly reported with two decimal places in both the main text and the figure itself.

(2). P values: We respectfully note that we have retained the original formatting for P values (e.g., P = 0.001 or P < 0.001) without converting them to two decimal places. We believe this is the standard practice in biomedical reporting, as P values below certain thresholds (e.g., 0.001) are conventionally presented with their exact values or inequality signs to maintain statistical clarity and avoid misleading precision. We have kept this formatting consistent across all figures, tables, and the main text.

(3). Table 1 special variables: For certain variables in Table 1 (e.g., counts, proportions, or variables with inherent units that do not lend themselves to two-decimal-place representation), we have retained the original precision that best reflects the nature of the data. We believe that forcing these variables into a two-decimal format would be scientifically inappropriate and potentially misleading. We have, however, ensured that all numerical values within the same category or column share consistent formatting.

We have carefully reviewed all figures and tables to ensure that numerical values are now presented with the greatest possible consistency while respecting the statistical and scientific conventions for different types of data. We believe these revisions address the reviewer's concerns and enhance the overall clarity of our manuscript. We thank the reviewer again for this valuable suggestion, which has improved the quality of our presentation.

2. Overall manuscript formatting
Please carefully review the formatting of the entire manuscript to ensure consistency, including figure and table presentation, font style, spacing, and numerical formatting where applicable.
Answer: We thank the reviewer for the careful attention to the formatting details of our manuscript. We agree that overall formatting consistency is essential for readability and professional presentation. Accordingly, we have conducted a thorough review of the entire manuscript and have made the following revisions:

(1). Figure and table presentation: We have carefully reviewed all figures and tables to ensure consistent formatting. For continuous variables, numerical values are now uniformly presented with two decimal places where such precision is appropriate, including the AUC values in Figure 2. We have also verified that all figure axes, labels, legends, and table headers follow a consistent style throughout the manuscript.

(2). Font style and spacing: We have standardized the font style, size, and line spacing across the entire manuscript, including the main text, figure legends, table footnotes, and supplementary materials, to ensure a uniform appearance.

(3). Numerical formatting: In addition to the two-decimal standard for continuous variables, we have standardized the formatting of P values, confidence intervals, and other statistical metrics throughout the text, tables, and figures. For P values, we have retained conventional reporting formats (e.g., exact values such as P = 0.001 or inequality notations such as P < 0.001), as converting these to two decimal places would be statistically inappropriate and potentially misleading. We have ensured that this convention is applied consistently across all sections.

We believe these revisions have substantially improved the overall consistency and readability of our manuscript. We are grateful to the reviewer for this constructive suggestion, which has undoubtedly enhanced the quality of our presentation.

3. Reference citation
Please revise the citation format throughout the manuscript. In particular, the newly added paragraph describing the biological functions of CHI3L1 should include appropriately placed in-text citations. References should be cited immediately after the corresponding statements they support, rather than listing multiple references only at the end of a long paragraph, to improve clarity and accurately attribute the supporting evidence.

Answer: We thank the reviewer for this valuable suggestion regarding the placement and formatting of in-text citations. We agree that proper citation placement is essential for accurately attributing supporting evidence and improving the clarity of the manuscript. Accordingly, we have made the following revisions:

(1). CHI3L1-related citations: We have carefully revised the newly added paragraph describing the biological functions of CHI3L1. Specifically, we have inserted appropriate in-text citations immediately after each corresponding statement or finding they support, rather than listing multiple references only at the end of the paragraph. This ensures that each specific claim is directly attributable to its original source, which we agree substantially improves the clarity and scientific rigor of the presentation.

(2). Review of all other citations: In addition to the CHI3L1 paragraph, we have thoroughly reviewed the entire manuscript and have revised the citation format throughout to ensure that all references are placed immediately after the statements they support. We have also verified that no long paragraphs end with a cluster of unspecific citations and that each reference is appropriately and accurately attributed.

We are grateful to the reviewer for this constructive feedback, which has helped us improve the accuracy and readability of our references and the overall quality of the manuscript.
