# Supplementary material for: Superior diagnostic efficacy and cost advantage of FibroTouch over CHI3L1 for liver fibrosis in Chinese patients with chronic liver disease
Source: PLoS One. 2026 Oct 5;21(10):e0357883. doi: 10.1371/journal.pone.0357883 (PMC13637865; doi:10.1371/journal.pone.0357883)
Supplement: Peer review history

# Peer review: Decision Letter 0

**DOI:** 10.1371/journal.pone.0357883.r001

**Authors:** Akif Altınbas

12 Jul 2026

Dear Dr. He,

Please submit your revised manuscript by Aug 26 2026 11:59PM. If you will need more time than this to complete your revisions, please reply to this message or contact the journal office at plosone@plos.org. When you're ready to submit your revision, log on to https://www.editorialmanager.com/pone/ and select the 'Submissions Needing Revision' folder to locate your manuscript file.

- A letter that responds to each point raised by the academic editor and reviewer(s). You should upload this letter as a separate file labeled 'Response to Reviewers'.
- A marked-up copy of your manuscript that highlights changes made to the original version. You should upload this as a separate file labeled 'Revised Manuscript with Track Changes'.
- An unmarked version of your revised paper without tracked changes. You should upload this as a separate file labeled 'Manuscript'.

If you would like to make changes to your financial disclosure, please include your updated statement in your cover letter. Guidelines for resubmitting your figure files are available below the reviewer comments at the end of this letter.

If applicable, we recommend that you deposit your laboratory protocols in protocols.io to enhance the reproducibility of your results. Protocols.io assigns your protocol its own identifier (DOI) so that it can be cited independently in the future. For instructions see: https://journals.plos.org/plosone/s/submission-guidelines#loc-laboratory-protocols. Additionally, PLOS ONE offers an option for publishing peer-reviewed Lab Protocol articles, which describe protocols hosted on protocols.io. Read more information on sharing protocols at https://plos.org/protocols?utm_medium=editorial-email&utm_source=authorletters&utm_campaign=protocols.

As the corresponding author, your ORCID iD is verified in the submission system and will appear in the published article. PLOS supports the use of ORCID, and we encourage all coauthors to register for an ORCID iD and use it as well. Please encourage your coauthors to verify their ORCID iD within the submission system before final acceptance, as unverified ORCID iDs will not appear in the published article. *Only* the individual author can complete the verification step; PLOS staff *cannot* verify ORCID iDs on behalf of authors.

We look forward to receiving your revised manuscript.

Kind regards,

Akif Altınbas, M.D.

Academic Editor

PLOS One

Journal Requirements:

When submitting your revision, we need you to address these additional requirements.

1. Please ensure that your manuscript meets PLOS ONE's style requirements, including those for file naming. The PLOS ONE style templates can be found at

https://journals.plos.org/plosone/s/file?id=wjVg/PLOSOne_formatting_sample_main_body.pdf and

https://journals.plos.org/plosone/s/file?id=ba62/PLOSOne_formatting_sample_title_authors_affiliations.pdf.

2. In the online submission form you indicate that your data is not available for proprietary reasons and have provided a contact point for accessing this data. Please note that your current contact point is a co-author on this manuscript. According to our Data Policy, the contact point must not be an author on the manuscript and must be an institutional contact, ideally not an individual. Please revise your data statement to a non-author institutional point of contact, such as a data access or ethics committee, and send this to us via return email. Please also include contact information for the third party organization, and please include the full citation of where the data can be found.

3. PLOS requires an ORCID iD for the corresponding author in Editorial Manager on papers submitted after December 6th, 2016. Please ensure that you have an ORCID iD and that it is validated in Editorial Manager. To do this, go to ‘Update my Information’ (in the upper left-hand corner of the main menu), and click on the Fetch/Validate link next to the ORCID field. This will take you to the ORCID site and allow you to create a new iD or authenticate a pre-existing iD in Editorial Manager.

4. Please be informed that funding information should not appear in the Acknowledgments section or other areas of your manuscript. We will only publish funding information present in the Funding Statement section of the online submission form. Please remove any funding-related text from the manuscript.

5. Your ethics statement should only appear in the Methods section of your manuscript. If your ethics statement is written in any section besides the Methods, please delete it from any other section.

6. If the reviewer comments include a recommendation to cite specific previously published works, please review and evaluate these publications to determine whether they are relevant and should be cited. There is no requirement to cite these works unless the editor has indicated otherwise.

[Note: HTML markup is below. Please do not edit.]

Reviewers' comments:

Reviewer's Responses to Questions

**Comments to the Author**

1. Is the manuscript technically sound, and do the data support the conclusions?

Reviewer #1: Yes

Reviewer #2: Partly

**********

2. Has the statistical analysis been performed appropriately and rigorously?

Reviewer #1: Yes

Reviewer #2: Yes

**********

3. Have the authors made all data underlying the findings in their manuscript fully available?

The PLOS Data policy

Reviewer #1: Yes

Reviewer #2: Yes

**********

4. Is the manuscript presented in an intelligible fashion and written in standard English?

Reviewer #1: Yes

Reviewer #2: Yes

**********

Reviewer #1: This manuscript focuses on comparing the diagnostic performance and economic benefits between FibroTouch and CHI3L1 for liver fibrosis assessment in Chinese patients with chronic liver disease. The research topic is clinically meaningful, and the sample size is acceptable with liver biopsy as the gold standard. However, multiple major flaws exist in study design, statistical analysis, data consistency, experimental quality control, presentation of results and manuscript writing. Therefore, a Major Revision is required. The detailed comments are listed as follows:

1. Patients undergoing liver biopsy often have more severe or "suspicious" symptoms, which might lead to an overrepresentation of advanced fibrosis (53.2% ≥S2). Please discuss how this prevalence affects the predictive values compared to a general screening population.

2.The manuscript states 235 enrolled patients in the main text, while the ROC curve legend marks the AUC value of LSM as 0.856 for significant fibrosis (≥S2), which conflicts with the text result of 0.850. All inconsistent numerical results need unified revision and verification.

3.The study adopted multiple comparison analyses among different fibrosis stages, but it only mentions using One-Way ANOVA and Dunn’s test in figure legends. The detailed application conditions and statistical results of multiple comparison tests are not supplemented in the Methods or Results section.

4.The authors conclude that FibroTouch (LSM) is superior to CHI3L1 and serological models based on AUROC values, but no pairwise comparison of ROC curves (such as DeLong’s test) was performed. It is impossible to confirm whether the differences in diagnostic efficacy are statistically significant.

5.While AUROC values are provided, the optimal cut-off values, sensitivity, specificity, positive predictive value (PPV), and negative predictive value (NPV) for each stage (≥S2, ≥S3, S4) are missing. These parameters are crucial for clinical decision-making. Please add a summary table for these diagnostic metrics.

6.The authors briefly mention single-center and retrospective limitations, but fail to further analyze the influence of these limitations on clinical application and result extrapolation. Meanwhile, potential confounding factors affecting CHI3L1 and LSM detection results are not discussed.

7.There are multiple grammatical and spelling errors in the full text. For example, "no competing interests exits" should be revised to "no competing interests exist"; the expression "Diagnostic of FibroTouch" in references is grammatically wrong. In addition, abbreviations are not standardized in some places.

8.The manuscript claims that LSM has an obvious cost advantage over CHI3L1, but only gives a qualitative description of "more than twice the cost". The specific charging standard, medical economic analysis and applicable scenarios are not supplemented.

9. Figure 2 has unclear label marking and incomplete group annotation; Table 1 arranges multiple indicators in a compact layout, and the grouping logic of partial data is confusing. The authors need to optimize the layout and supplement detailed legends.

10.Why were these specific six models (especially FIB-5 and PRP) chosen as comparators? Some, like FIB-5, showed very poor performance (AUROC < 0.6). It would be helpful to justify their relevance or compare with more widely accepted tools like the Enhanced Liver Fibrosis (ELF) score if available.

Reviewer #2: In this manuscript, the authors evaluated the diagnostic performance of serum CHI3L1 as a non-invasive biomarker for liver fibrosis in patients with chronic liver disease. CHI3L1 was compared with liver stiffness measurement (LSM) and several established serological fibrosis models. Overall, the study addresses a clinically relevant topic and is generally well organized. However, several issues should be addressed to strengthen the manuscript.

1. Introduction: The Introduction should provide a more comprehensive overview of CHI3L1. Specifically, the authors should describe the major cellular sources of CHI3L1, the physiological and pathological conditions under which it is produced, and the biological rationale for its use as a biomarker of liver fibrosis.

2. Sample size and external validation: The study cohort is relatively small (n = 235), which may limit the generalizability of the findings. The authors are encouraged to validate their results in an independent cohort or a publicly available dataset to further support the robustness of their conclusions.

3. Table 1: Please clarify what diseases are included in the "OTHERS" category in Table 1. Since CHI3L1 expression may vary among different etiologies of chronic liver disease, the composition of this group should be clearly defined.

4. Section 3.4: The diagnostic performance is mainly described in the text. For improved readability, the corresponding statistical results, including AUROC, 95% confidence intervals, P values, sensitivity, specificity, and optimal cutoff values (where applicable), should be summarized in a table or figure.

5. Validation of fibrosis staging: The current analyses mainly evaluate the ability of CHI3L1 and LSM to identify different fibrosis thresholds (e.g., ≥S2, ≥S3, and S4). However, it remains unclear whether these biomarkers can reliably discriminate individual fibrosis stages. The authors are encouraged to perform additional analyses, such as pairwise comparisons between adjacent fibrosis stages or multiclass classification analyses, to evaluate their ability to distinguish each fibrosis stage. These additional analyses would further support the clinical utility of CHI3L1 for fibrosis staging.

**********

what does this mean?). If published, this will include your full peer review and any attached files.

If you choose “no”, your identity will remain anonymous but your review may still be made public.

**Do you want your identity to be public for this peer review?** For information about this choice, including consent withdrawal, please see our Privacy Policy

Reviewer #1: **Yes:**  Yanqiu Huang

Reviewer #2: No

**********

[NOTE: If reviewer comments were submitted as an attachment file, they will be attached to this email and accessible via the submission site. Please log into your account, locate the manuscript record, and check for the action link "View Attachments". If this link does not appear, there are no attachment files.]

To ensure your figures meet our technical requirements, please review our figure guidelines: https://journals.plos.org/plosone/s/figures

You may also use PLOS’s free figure tool, NAAS, to help you prepare publication quality figures: https://journals.plos.org/plosone/s/figures#loc-tools-for-figure-preparation.

NAAS will assess whether your figures meet our technical requirements by comparing each figure against our figure specifications.

Attachment Submitted filename: Review Report.pdf

# Author comment: Author response to Decision Letter 1

**DOI:** 10.1371/journal.pone.0357883.r002

28 Jul 2026

The detailed information were in the Word document "Response to Reviewer1/2" detached in the system. We greatly appreciate your suggestion, which has substantially improved the statistical rigor of our study.

Attachment Submitted filename: response to reviewer.docx

# Peer review: Decision Letter 1

**DOI:** 10.1371/journal.pone.0357883.r003

**Authors:** Hamidreza Karimi-Sari

19 Aug 2026

Dear Dr. He,

Thank you for submitting your manuscript to PLOS One. After careful consideration, we feel that it has merit but does not fully meet PLOS One’s publication criteria as it currently stands. Therefore, we invite you to submit a revised version of the manuscript that addresses the points raised during the review process.

Please submit your revised manuscript by Oct 03 2026 11:59PM. If you will need more time than this to complete your revisions, please reply to this message or contact the journal office at plosone@plos.org. When you're ready to submit your revision, log on to https://www.editorialmanager.com/pone/ and select the 'Submissions Needing Revision' folder to locate your manuscript file.

- A letter that responds to each point raised by the academic editor and reviewer(s). You should upload this letter as a separate file labeled 'Response to Reviewers'.
- A marked-up copy of your manuscript that highlights changes made to the original version. You should upload this as a separate file labeled 'Revised Manuscript with Track Changes'.
- An unmarked version of your revised paper without tracked changes. You should upload this as a separate file labeled 'Manuscript'.

If you would like to make changes to your financial disclosure, please include your updated statement in your cover letter. Guidelines for resubmitting your figure files are available below the reviewer comments at the end of this letter.

If applicable, we recommend that you deposit your laboratory protocols in protocols.io to enhance the reproducibility of your results. Protocols.io assigns your protocol its own identifier (DOI) so that it can be cited independently in the future. For instructions see: https://journals.plos.org/plosone/s/submission-guidelines#loc-laboratory-protocols. Additionally, PLOS One offers an option for publishing peer-reviewed Lab Protocol articles, which describe protocols hosted on protocols.io. Read more information on sharing protocols at https://plos.org/protocols?utm_medium=editorial-email&utm_source=authorletters&utm_campaign=protocols.

As the corresponding author, your ORCID iD is verified in the submission system and will appear in the published article. PLOS supports the use of ORCID, and we encourage all coauthors to register for an ORCID iD and use it as well. Please encourage your coauthors to verify their ORCID iD within the submission system before final acceptance, as unverified ORCID iDs will not appear in the published article. *Only* the individual author can complete the verification step; PLOS staff *cannot* verify ORCID iDs on behalf of authors.

We look forward to receiving your revised manuscript.

Kind regards,

Hamidreza Karimi-Sari

Academic Editor

PLOS One

Journal Requirements:

If the reviewer comments include a recommendation to cite specific previously published works, please review and evaluate these publications to determine whether they are relevant and should be cited. There is no requirement to cite these works unless the editor has indicated otherwise.

Please review your reference list to ensure that it is complete and correct. If you have cited papers that have been retracted, please include the rationale for doing so in the manuscript text, or remove these references and replace them with relevant current references. Any changes to the reference list should be mentioned in the rebuttal letter that accompanies your revised manuscript. If you need to cite a retracted article, indicate the article’s retracted status in the References list and also include a citation and full reference for the retraction notice.

[Note: HTML markup is below. Please do not edit.]

Reviewers' comments:

Reviewer's Responses to Questions

**Comments to the Author**

Reviewer #2: All comments have been addressed

**********

2. Is the manuscript technically sound, and do the data support the conclusions?

Reviewer #2: Yes

**********

3. Has the statistical analysis been performed appropriately and rigorously?

Reviewer #2: Yes

**********

4. Have the authors made all data underlying the findings in their manuscript fully available?

The PLOS Data policy

Reviewer #2: Yes

**********

5. Is the manuscript presented in an intelligible fashion and written in standard English?

Reviewer #2: Yes

**********

Reviewer #2: 1. Figure formatting

Please improve the consistency of figure formatting. For example, in Supplementary Figures S1 and S2, numerical values are reported with inconsistent decimal precision (e.g., some values are presented with two decimal places, whereas others are reported with four decimal places). Please standardize the numerical formatting throughout all figures and tables.

2. Overall manuscript formatting

Please carefully review the formatting of the entire manuscript to ensure consistency, including figure and table presentation, font style, spacing, and numerical formatting where applicable.

3. Reference citation

Please revise the citation format throughout the manuscript. In particular, the newly added paragraph describing the biological functions of CHI3L1 should include appropriately placed in-text citations. References should be cited immediately after the corresponding statements they support, rather than listing multiple references only at the end of a long paragraph, to improve clarity and accurately attribute the supporting evidence.

**********

what does this mean?). If published, this will include your full peer review and any attached files.

If you choose “no”, your identity will remain anonymous but your review may still be made public.

**Do you want your identity to be public for this peer review?** For information about this choice, including consent withdrawal, please see our Privacy Policy

Reviewer #2: No

**********

[NOTE: If reviewer comments were submitted as an attachment file, they will be attached to this email and accessible via the submission site. Please log into your account, locate the manuscript record, and check for the action link "View Attachments". If this link does not appear, there are no attachment files.]

To ensure your figures meet our technical requirements, please review our figure guidelines: https://journals.plos.org/plosone/s/figures

You may also use PLOS’s free figure tool, NAAS, to help you prepare publication quality figures: https://journals.plos.org/plosone/s/figures#loc-tools-for-figure-preparation.

NAAS will assess whether your figures meet our technical requirements by comparing each figure against our figure specifications.

# Author comment: Author response to Decision Letter 2

**DOI:** 10.1371/journal.pone.0357883.r004

22 Aug 2026

Reviewer #2:

1. Figure formatting

Please improve the consistency of figure formatting. For example, in Supplementary Figures S1 and S2, numerical values are reported with inconsistent decimal precision (e.g., some values are presented with two decimal places, whereas others are reported with four decimal places). Please standardize the numerical formatting throughout all figures and tables.

Answer: We thank the reviewer for the careful attention to the formatting details of our figures and tables. We agree that consistency in numerical presentation is important for clarity and have thoroughly revised all figures and tables accordingly.

Specifically, we have made the following changes:

(1). Figures and Tables: We have standardized the numerical formatting throughout all figures and tables ((including Supplementary Table S1 and S2)) to two decimal places for all continuous variables where such precision is appropriate. This includes the AUC values presented in Figure 2, which have now been uniformly reported with two decimal places in both the main text and the figure itself.

(2). P values: We respectfully note that we have retained the original formatting for P values (e.g., P = 0.001 or P < 0.001) without converting them to two decimal places. We believe this is the standard practice in biomedical reporting, as P values below certain thresholds (e.g., 0.001) are conventionally presented with their exact values or inequality signs to maintain statistical clarity and avoid misleading precision. We have kept this formatting consistent across all figures, tables, and the main text.

(3). Table 1 special variables: For certain variables in Table 1 (e.g., counts, proportions, or variables with inherent units that do not lend themselves to two-decimal-place representation), we have retained the original precision that best reflects the nature of the data. We believe that forcing these variables into a two-decimal format would be scientifically inappropriate and potentially misleading. We have, however, ensured that all numerical values within the same category or column share consistent formatting.

We have carefully reviewed all figures and tables to ensure that numerical values are now presented with the greatest possible consistency while respecting the statistical and scientific conventions for different types of data. We believe these revisions address the reviewer's concerns and enhance the overall clarity of our manuscript. We thank the reviewer again for this valuable suggestion, which has improved the quality of our presentation.

2. Overall manuscript formatting

Please carefully review the formatting of the entire manuscript to ensure consistency, including figure and table presentation, font style, spacing, and numerical formatting where applicable.

Answer: We thank the reviewer for the careful attention to the formatting details of our manuscript. We agree that overall formatting consistency is essential for readability and professional presentation. Accordingly, we have conducted a thorough review of the entire manuscript and have made the following revisions:

(1). Figure and table presentation: We have carefully reviewed all figures and tables to ensure consistent formatting. For continuous variables, numerical values are now uniformly presented with two decimal places where such precision is appropriate, including the AUC values in Figure 2. We have also verified that all figure axes, labels, legends, and table headers follow a consistent style throughout the manuscript.

(2). Font style and spacing: We have standardized the font style, size, and line spacing across the entire manuscript, including the main text, figure legends, table footnotes, and supplementary materials, to ensure a uniform appearance.

(3). Numerical formatting: In addition to the two-decimal standard for continuous variables, we have standardized the formatting of P values, confidence intervals, and other statistical metrics throughout the text, tables, and figures. For P values, we have retained conventional reporting formats (e.g., exact values such as P = 0.001 or inequality notations such as P < 0.001), as converting these to two decimal places would be statistically inappropriate and potentially misleading. We have ensured that this convention is applied consistently across all sections.

We believe these revisions have substantially improved the overall consistency and readability of our manuscript. We are grateful to the reviewer for this constructive suggestion, which has undoubtedly enhanced the quality of our presentation.

3. Reference citation

Please revise the citation format throughout the manuscript. In particular, the newly added paragraph describing the biological functions of CHI3L1 should include appropriately placed in-text citations. References should be cited immediately after the corresponding statements they support, rather than listing multiple references only at the end of a long paragraph, to improve clarity and accurately attribute the supporting evidence.

Answer: We thank the reviewer for this valuable suggestion regarding the placement and formatting of in-text citations. We agree that proper citation placement is essential for accurately attributing supporting evidence and improving the clarity of the manuscript. Accordingly, we have made the following revisions:

(1). CHI3L1-related citations: We have carefully revised the newly added paragraph describing the biological functions of CHI3L1. Specifically, we have inserted appropriate in-text citations immediately after each corresponding statement or finding they support, rather than listing multiple references only at the end of the paragraph. This ensures that each specific claim is directly attributable to its original source, which we agree substantially improves the clarity and scientific rigor of the presentation.

(2). Review of all other citations: In addition to the CHI3L1 paragraph, we have thoroughly reviewed the entire manuscript and have revised the citation format throughout to ensure that all references are placed immediately after the statements they support. We have also verified that no long paragraphs end with a cluster of unspecific citations and that each reference is appropriately and accurately attributed.

We are grateful to the reviewer for this constructive feedback, which has helped us improve the accuracy and readability of our references and the overall quality of the manuscript.

Attachment Submitted filename: response to Review 2.docx

# Editor report: Decision Letter 2

**DOI:** 10.1371/journal.pone.0357883.r005

**Authors:** Hamidreza Karimi-Sari

24 Aug 2026

<p>Superior Diagnostic Efficacy and Cost Advantage of FibroTouch Over CHI3L1 for Liver Fibrosis in Chinese Patients with Chronic Liver Disease

PONE-D-26-19383R2

Dear Dr. He,

We’re pleased to inform you that your manuscript has been judged scientifically suitable for publication and will be formally accepted for publication once it meets all outstanding technical requirements.

Within one week, you’ll receive an e-mail detailing the required amendments. When these have been addressed, you’ll receive a formal acceptance letter and your manuscript will be scheduled for publication.

An invoice will be generated when your article is formally accepted. Please note, if your institution has a publishing partnership with PLOS and your article meets the relevant criteria, all or part of your publication costs will be covered. Please make sure your user information is up-to-date by logging into Editorial Manager at Editorial Manager® and clicking the ‘Update My Information' link at the top of the page. For questions related to billing, please contact billing support.

If your institution or institutions have a press office, please notify them about your upcoming paper to help maximize its impact. If they’ll be preparing press materials, please inform our press team as soon as possible -- no later than 48 hours after receiving the formal acceptance. Your manuscript will remain under strict press embargo until 2 pm Eastern Time on the date of publication. For more information, please contact onepress@plos.org.

Kind regards,

Hamidreza Karimi-Sari

Academic Editor

PLOS One

Additional Editor Comments (optional):

Reviewers' comments:

# Editor report: Acceptance letter

**DOI:** 10.1371/journal.pone.0357883.r006

**Authors:** Hamidreza Karimi-Sari

PONE-D-26-19383R2

PLOS One

Dear Dr. He,

I'm pleased to inform you that your manuscript has been deemed suitable for publication in PLOS One. Congratulations! Your manuscript is now being handed over to our production team.

At this stage, our production department will prepare your paper for publication. This includes ensuring the following:

* All references, tables, and figures are properly cited

* All relevant supporting information is included in the manuscript submission,

* There are no issues that prevent the paper from being properly typeset

You will receive further instructions from the production team, including instructions on how to review your proof when it is ready. Please keep in mind that we are working through a large volume of accepted articles, so please give us a few days to review your paper and let you know the next and final steps.

Lastly, if your institution or institutions have a press office, please let them know about your upcoming paper now to help maximize its impact. If they'll be preparing press materials, please inform our press team within the next 48 hours. Your manuscript will remain under strict press embargo until 2 pm Eastern Time on the date of publication. For more information, please contact onepress@plos.org.

You will receive an invoice from PLOS for your publication fee after your manuscript has reached the completed accept phase. If you receive an email requesting payment before acceptance or for any other service, this may be a phishing scheme. Learn how to identify phishing emails and protect your accounts at https://explore.plos.org/phishing.

If we can help with anything else, please email us at customercare@plos.org.

Thank you for submitting your work to PLOS One and supporting open access.

Kind regards,

PLOS One Editorial Office Staff

on behalf of

Dr. Hamidreza Karimi-Sari

Academic Editor

PLOS One
